# Global trends and projections of rehabilitation needs in chronic obstructive pulmonary disease, 1990–2050: an analysis using WHO rehabilitation need estimates

**DOI:** 10.3389/fpubh.2026.1921263

**Published:** 2026-09-14

**Authors:** Yu Qiu, Yue Lan, Yan Hou, Xin Huang, Ling Huang, Junjie Xia

**Affiliations:** 1 Department of Respiratory and Critical Care Medicine, The Third Hospital of Mianyang/Sichuan Mental Health Center, Mianyang, Sichuan, China; 2 Department of Respiratory and Critical Care Medicine, The Affiliated Hospital of Southwest Medical University, Luzhou, Sichuan, China

**Keywords:** chronic obstructive pulmonary disease, health inequality, pulmonary rehabilitation, rehabilitation need, years lived with disability

## Abstract

**Background:**

Chronic obstructive pulmonary disease (COPD) is a major contributor to disability worldwide. Rehabilitation plays an important role in improving functional outcomes, yet global patterns of potential population-level rehabilitation needs among people with COPD remain insufficiently described.

**Methods:**

We used the WHO Rehabilitation Need Estimates to estimate potential rehabilitation needs and years lived with disability (YLDs) among people with COPD across 195 countries from 1990 to 2021, with projections to 2050. Temporal trends, decomposition of drivers, associations with the socio-demographic index (SDI), and inequality indices were assessed.

**Results:**

In 2021, potential rehabilitation needs related to COPD reached 123.5 million (95% UI 105.5–142.2), and YLDs reached 14.9 million (11.9–17.9). From 1990 to 2021, both measures increased substantially, whereas the age-standardized prevalence rate (ASPR) remained stable and the age-standardized YLD rate (ASYR) increased slightly. The largest burdens were observed in the South-East Asia and Western Pacific Regions, with the fastest growth in the African and Eastern Mediterranean Regions. China, India, and the United States had the largest absolute numbers. By 2050, potential rehabilitation needs and YLDs are projected to reach 150.2 million and 18.6 million, respectively. Increases were mainly driven by population growth and population aging. Women had higher absolute numbers of potential rehabilitation needs and YLDs, whereas men had higher ASPRs at older ages. YLDs were disproportionately concentrated in lower-SDI countries, with persistent inequalities.

**Conclusion:**

Potential rehabilitation needs among people with COPD have increased substantially and are projected to continue rising through 2050, primarily driven by demographic changes and persistent socioeconomic inequalities. These findings highlight the importance of strengthening COPD prevention and improving rehabilitation planning, particularly for aging populations and lower-SDI settings.

## 1 Introduction

Chronic obstructive pulmonary disease (COPD) remains one of the leading contributors to global disease burden. The global prevalence of COPD is estimated at 10.3% (1, 2), affecting hundreds of millions of individuals and imposing substantial health and socioeconomic burdens. COPD is associated with disabling symptoms such as dyspnea, fatigue, anxiety, and depression, which impair daily functioning and quality of life while increasing the risks of hospitalization and premature death (3, 4). Between 1990 and 2021, disability-adjusted life years (DALYs) due to COPD increased by 40.3%, from 56.9 million to 79.8 million (5), reflecting the growing impact of the disease globally. This increasing burden highlights the importance of effective long-term management strategies, including rehabilitation as an important component of comprehensive COPD management.

Pulmonary rehabilitation (PR) is an effective intervention in COPD management and has been shown to alleviate symptoms, improve exercise tolerance and health-related quality of life (HRQoL), and reduce hospitalizations and post-discharge mortality (6–10). Consequently, a substantial proportion of individuals with COPD may benefit from rehabilitation services throughout the course of their disease. However, population aging and the increasing burden of COPD are expected to increase potential population-level rehabilitation needs worldwide. Despite these potential needs, access to rehabilitation services remains limited in many settings because of factors including limited resources, inadequate policy prioritization, and barriers related to accessibility and cost (11). Furthermore, the magnitude, geographic distribution, future trends, and socioeconomic inequalities of potential rehabilitation needs among people with COPD have not been comprehensively evaluated at the global level.

To address this knowledge gap, we used the World Health Organization (WHO) Rehabilitation Need Estimates (12) to comprehensively assess potential rehabilitation needs among people with COPD and years lived with disability (YLDs) at global, regional, and national levels from 1990 to 2021 and to project future trends through 2050. We further quantified the contributions of demographic and epidemiological drivers to changes in potential rehabilitation needs and evaluated socioeconomic inequalities across different levels of development. By characterizing temporal trends, future trajectories, and disparities in potential rehabilitation needs, this study provides evidence to support public health planning, priority setting, and rehabilitation system planning.

## 2 Methods

### 2.1 Data sources and definitions

We obtained data from the WHO Rehabilitation Need Estimates, which provide estimates of prevalence and YLDs for 27 diseases, impairments, and sequelae considered amenable to rehabilitation. These estimates represent potential population-level rehabilitation needs, referring to populations who may benefit from rehabilitation interventions rather than actual rehabilitation demand. For COPD, we extracted data from 1990 to 2021 at global, regional, and national levels across 195 countries, including individuals aged ≥15 years grouped into five-year age intervals up to 95 + years and stratified by sex (male, female, and both sexes). Regional estimates followed the classification system used in the WHO Rehabilitation Need Estimates database, including WHO geographic regions and World Bank income groups. World Bank High Income represents an income-based grouping rather than a WHO geographic region. Indicators included prevalence estimates, YLDs, and their corresponding rates per 100,000 population.

COPD case definitions in the WHO Rehabilitation Need Estimates were primarily based on the Global Initiative for Chronic Obstructive Lung Disease (GOLD) criteria. COPD was identified using post-bronchodilator spirometry with a forced expiratory volume in 1 s to forced vital capacity ratio (FEV_1_/FVC) of less than 0.7 as the diagnostic threshold (3). Cases were mapped using International Classification of Diseases (ICD)-10 codes J41–J44 and J47 and ICD-9 codes 491–492 and 496. To improve comparability across heterogeneous epidemiological data sources, the WHO Rehabilitation Need Estimates also incorporated evidence based on alternative diagnostic definitions, including GOLD pre-bronchodilation, Lower Limit of Normal (LLN)-based definitions, and the European Respiratory Society (ERS) guidelines (13).

In the WHO Rehabilitation Need Estimates, prevalence and YLDs are reported for health conditions considered amenable to rehabilitation and are used to estimate potential rehabilitation needs. Accordingly, COPD prevalence estimates and ASPRs were used as population-level indicators of potential rehabilitation needs, whereas YLDs and ASYRs were used to reflect the associated burden of disability and functional impairment (12, 13). However, these estimates do not capture individual-level disease severity, functional limitation, clinical characteristics, or rehabilitation service availability.

### 2.2 Statistical analysis

Age-standardized prevalence rates (ASPR) and age-standardized YLD rates (ASYR) for COPD were obtained directly from the WHO Rehabilitation Need Estimates. According to the database methodology, age-standardized rates (ASRs) were calculated using the Global Burden of Disease 2021 Study (GBD 2021) standard population as: $\mathrm{ASR}=\frac{\sum_{i=1}^{k}a_{i}\;w_{i}}{\sum_{i=1}^{k}w_{i}}$ , where *a_i_* is the age-specific rate for the *i*-th age group, *w_i_* is the weight in the GBD 2021 standard population, and *k* is the number of age groups. All estimates were derived from 1,000 posterior draws provided in the WHO Rehabilitation Need Estimates, with 95% uncertainty intervals (UIs) defined by the 2.5th and 97.5th percentiles (14).

We calculated the estimated annual percentage change (EAPC) to assess temporal trends in ASPR and ASYR for COPD from 1990 to 2021, using a log-linear regression model: $\ln(\mathrm{AS}R_{t})=\alpha +\mathrm{\beta t}+\epsilon$ , where t represents calendar year (1990–2021) and β is the slope. EAPC and its 95% confidence interval (CI) were calculated as: $\text{EAPC}=100\times (e^{\beta }-1),\text{and}\;\;{\text{EAPC}}_{\text{lower}/\text{upper}}=100\times (e^{\left(\beta \pm 1.96\times \mathrm{SE}\right)}-1),$ where SE is the standard error of β. A trend was considered significant if the 95% CI excluded zero. Positive EAPC values indicated an increasing trend, negative values a decreasing trend, and values with CIs including zero indicated no significant change (15).

To estimate future trends as plausible scenarios based on historical patterns, we fitted Bayesian age–period–cohort (BAPC) models to the 1990–2021 WHO Rehabilitation Need Estimates and projected potential rehabilitation needs related to COPD and YLDs, including their corresponding ASPR and ASYR, from 2022 to 2050. Bayesian inference was implemented through Integrated Nested Laplace Approximation (INLA) (16) to estimate age, period, and cohort effects. The projected estimates and uncertainty intervals were obtained from the posterior distributions of the fitted BAPC models.

To identify the main drivers of change, we decomposed the total increase in COPD potential rehabilitation needs and YLDs between 1990 and 2021 into three components: population growth, population aging, and epidemiological change. Population growth reflected changes in population size, population aging reflected shifts in age structure, and epidemiological change represented changes beyond demographic effects, including variations in disease patterns and other non-demographic factors. Interaction effects between these components were proportionally redistributed among the three components (17, 18).

The Socio-demographic Index (SDI), a composite index based on education, fertility, and income with values ranging from 0 to 1 (19), was used to assess associations between SDI and ASPR and ASYR. Countries were ranked by population-weighted SDI and classified into quintiles according to the GBD 2021 framework. At the national level, Spearman’s rank correlation was used to examine associations between SDI and ASPR and ASYR.

To further quantify disparities across SDI strata, we performed inequality analyses based on absolute and relative measures. We assessed inequality using the slope index of inequality (SII) and the concentration index of inequality (CII). These metrics were computed for ASRs of potential rehabilitation needs and YLDs across SDI quintiles from 1990 to 2021 to quantify both absolute (SII) and relative (CII) inequalities. Positive estimates suggested a greater burden in higher-SDI settings, negative estimates suggested a greater burden in lower-SDI settings, and values near zero indicated little difference in burden across countries (20, 21).

Analytical details are provided in Supplementary material. All analyses and visualizations were performed using R software (version 4.4.2; R Foundation for Statistical Computing, Vienna, Austria) and RStudio (version 2023.6.1.524; Posit PBC, Boston, MA, United States). Statistical significance was defined as a two-sided *p* < 0.05.

## 3 Results

### 3.1 Global potential rehabilitation needs

In 2021, an estimated 123.5 million (95% UI 105.5–142.2) people with COPD had potential rehabilitation needs, representing a 2.2-fold increase in the absolute number compared with 57.3 million (48.8–66.0) in 1990. Over the same period, YLDs due to COPD increased from 6.8 million (5.4–8.2) to 14.9 million (11.9–17.9), representing a 2.2-fold increase. Despite this substantial increase in the absolute number of potential rehabilitation needs, the corresponding ASPR remained stable at 2036.7 (1742.3–2341.8) per 100,000 population in 2021 (EAPC 0.03, 95% CI − 0.02 to 0.07). In contrast, ASYR increased significantly to 244.5 (196.0–293.6) per 100,000 population in 2021, with an EAPC of 0.13 (95% CI 0.07–0.19) (Table 1).

**Table 1:** Burden of COPD prevalence and YLDs in 1990 and 2021, and temporal trends from 1990 to 2021 at global and regional levels.

Location	Prevalence					YLDs				
	1990		2021			1990		2021		
	Number (95% UI)	ASR per 100,000 (95% UI)	Number (95% UI)	ASR per 100,000 (95% UI)	EAPC (95% CI)	Number (95% UI)	ASR per 100,000 (95% UI)	Number (95% UI)	ASR per 100,000 (95% UI)	EAPC (95% CI)
Global										
Total	57,326,411 (48,783,779–66,043,113)	2025.63 (1723.06–2334.12)	123,519,489 (105,453,632–142,248,133)	2036.68 (1742.28–2341.84)	0.03 (−0.02–0.07)	6,796,664 (5,436,303–8,162,583)	236.74 (189.65–284.16)	14,857,505 (11,884,328–17,859,742)	244.53 (196.04–293.56)	0.13 (0.07–0.19)
Male	27,286,999 (23,019,208–31,665,485)	2116.73 (1783.54–2457.96)	59,326,013 (50,454,649–68,698,082)	2113.83 (1798.21–2446.09)	−0.01 (−0.09 to 0.07)	3,057,026 (2,424,664–3,709,227)	232.31 (184.00–282.23)	6,727,440 (5,343,046–8,155,568)	237.81 (188.92–288.36)	0.09 (−0.01 to 0.20)
Female	30,039,412 (25,537,725–34,637,281)	1961.55 (1666.24–2264.39)	64,193,476 (54,774,979–73,972,187)	1974.29 (1684.30–2274.97)	0.05 (0.03–0.07)	3,739,638 (2,941,496–4,560,670)	241.89 (190.17–295.17)	8,130,065 (6,407,030–9,929,771)	250.63 (197.52–306.10)	0.14 (0.11–0.17)
Region										
African Region	5,506,699 (4,558,664–6,508,981)	1552.70 (1275.72–1848.01)	13,725,912 (11,444,046–16,205,017)	1685.22 (1399.21–1997.41)	0.22 (0.20–0.24)	778,970 (605,211–962,209)	216.57 (167.64–268.77)	1,933,352 (1,494,264–2,390,745)	232.63 (179.64–288.49)	0.20 (0.18–0.21)
Eastern Mediterranean Region	4,953,068 (4,115,789–5,802,651)	1937.48 (1602.06–2278.29)	12,951,926 (10,805,215–15,246,071)	2122.34 (1766.72–2502.53)	0.29 (0.28–0.30)	630,746 (492,241–777,492)	245.90 (191.38–303.95)	1,577,789 (1,235,669–1,942,031)	254.66 (199.43–313.68)	0.15 (0.12–0.19)
European Region	8,839,591 (7,322,646–10,479,966)	1671.40 (1382.05–1985.62)	12,583,865 (10,581,042–14,795,540)	1650.03 (1386.94–1939.05)	−0.07 (−0.09 to −0.05)	807,493 (634,553–1,000,287)	152.55 (119.74–189.26)	1,139,854 (908,227–1,397,874)	149.79 (119.28–183.87)	−0.11 (−0.14 to −0.08)
Region of the Americas	4,494,622 (3,644,230–5,438,424)	1258.43 (1019.38–1524.77)	12,474,669 (10,182,873–15,006,837)	1341.45 (1094.86–1613.61)	0.20 (0.16–0.23)	392,229 (304,675–488,674)	108.21 (84.17–134.78)	1,078,761 (841,486–1,344,022)	115.79 (90.37–144.24)	0.21 (0.18–0.24)
South-East Asia Region	26,418,505 (22,527,336–30,100,443)	2579.58 (2189.92–2944.94)	67,144,693 (57,293,504–76,601,441)	2638.56 (2252.33–3006.19)	0.06 (0.02–0.11)	3,655,398 (2,901,149–4,413,258)	352.60 (280.01–426.04)	9,302,941 (7,400,012–11,231,547)	363.50 (289.66–438.43)	0.13 (0.03–0.23)
Western Pacific Region	28,803,020 (24,211,106–33,317,834)	2160.39 (1807.68–2508.09)	63,686,260 (52,673,553–75,464,455)	1983.20 (1640.62–2346.27)	−0.27 (−0.31 to −0.22)	3,940,649 (3,074,271–4,810,944)	295.45 (229.51–361.87)	8,540,326 (6,567,614–10,569,423)	266.33 (205.01–329.74)	−0.32 (−0.36 to −0.28)
World Bank High Income	34,992,091 (29,793,615–40,517,239)	1913.24 (1627.20–2217.59)	62,973,694 (55,306,499–71,183,197)	1906.85 (1672.83–2157.41)	0.04 (−0.02 to 0.11)	3,330,043 (2,683,751–4,020,538)	182.43 (146.84–220.48)	6,021,615 (5,002,901–7,132,688)	184.00 (152.56–218.11)	0.09 (−0.00 to 0.18)

YLDs, years lived with disability; COPD, chronic obstructive pulmonary disease; ASR, age-standardized rate; EAPC, estimated annual percentage change; UI, uncertainty interval; CI, confidence interval.

### 3.2 Regional potential rehabilitation needs

In 2021, the South-East Asia Region had the highest number of people with potential rehabilitation needs (67.1 million), followed by Western Pacific Region (63.7 million) and World Bank High Income group (63.0 million), whereas the Region of the Americas had the lowest number (12.5 million). Between 1990 and 2021, potential rehabilitation needs increased in all regions. The largest relative increases were observed in the African Region and Eastern Mediterranean Region, where potential rehabilitation needs increased by approximately 2.5-fold (Table 1; Supplementary Figure S1A).

For COPD-related YLDs, the South-East Asia Region (9.3 million) and Western Pacific Region (8.5 million) recorded the highest values in 2021, followed by World Bank High Income group (6.0 million). YLDs increased across all regions from 1990 to 2021, with the most marked relative increases occurring in the Region of the Americas, Eastern Mediterranean Region, and African Region (Table 1; Supplementary Figure S2A).

For ASPR, the South-East Asia Region had the highest ASPR in 2021 (2638.6 per 100,000 population), followed by Eastern Mediterranean Region (2122.3 per 100,000 population) and Western Pacific Region (1983.2 per 100,000 population), whereas the Region of the Americas had the lowest ASPR (1341.5 per 100,000 population). ASPR increased in most regions during 1990–2021, with the largest increases observed in the Eastern Mediterranean Region (EAPC 0.29, 95% CI 0.28–0.30) and African Region (0.22, 0.20–0.24). Significant declines were observed only in the Western Pacific Region (−0.27, −0.31 to −0.22) and European Region (−0.07, −0.09 to −0.05) (Table 1; Supplementary Figure S1B).

A similar pattern was observed for ASYR. The South-East Asia Region (363.5 per 100,000 population) and Western Pacific Region (266.3 per 100,000 population) had the highest rates in 2021. During 1990–2021, ASYR decreased in the Western Pacific Region (−0.32, −0.36 to −0.28) and European Region (−0.11, −0.14 to −0.08), whereas the largest increases were observed in the Region of the Americas (0.21, 0.18–0.24) and African Region (0.20, 0.18–0.21) (Table 1; Supplementary Figure S2B).

### 3.3 National potential rehabilitation needs

At the national level, China, India, and the United States had the largest numbers of people with potential rehabilitation needs in 2021, with an estimated 28.5 (95% UI 23.4–33.9) million, 24.6 (20.9–28.0) million, and 12.2 (11.0–13.2) million people, respectively. In contrast, several small island states, including Niue, Nauru, and Tuvalu, had fewer than 200 people with potential rehabilitation needs (Supplementary Table S1; Figure 1A). A similar geographical pattern was observed for YLDs, with China (3.9 million, 3.0–4.8), India (3.5 million, 2.8–4.3), and the United States (1.3 million, 1.1–1.5) reporting the highest YLDs, whereas Niue, Nauru, and Tuvalu each recorded fewer than 20 YLDs (Supplementary Table S1; Figure 1B).

**Figure 1:**
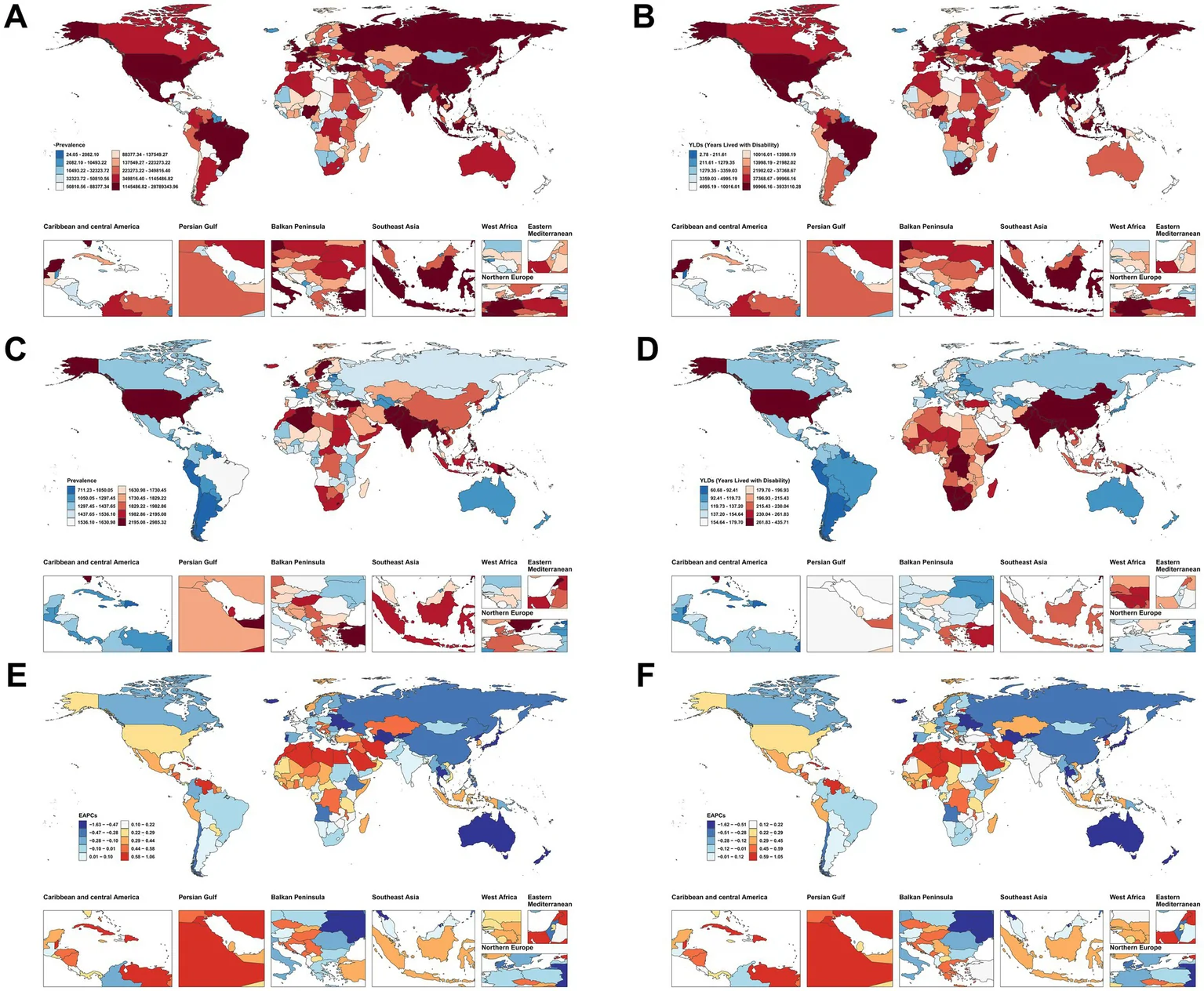
Numbers of prevalent cases **(A)** and years lived with disability (YLDs) **(B)**, age-standardized rates of prevalence **(C)** and YLDs **(D)** for chronic obstructive pulmonary disease (COPD) across countries and territories in 2021, and estimated annual percentage changes (EAPCs) in prevalence **(E)** and YLDs **(F)** from 1990 to 2021. Prevalence estimates were used as population-level indicators of potential rehabilitation needs.

The highest ASPRs in 2021 were observed in the United States (2955.8 per 100,000 population), Qatar (2936.2 per 100,000 population), and Nepal (2772.7 per 100,000 population), whereas Singapore, Chile, and Barbados had the lowest ASPRs, all below 800 per 100,000 population (Supplementary Table S1; Figure 1C). The highest ASYRs were observed in Nepal (431.4 per 100,000 population), India (416.7 per 100,000 population), and Pakistan (398.7 per 100,000 population), while Chile, Saint Vincent and the Grenadines, and Barbados had the lowest ASYRs (Supplementary Table S1; Figure 1D).

From 1990 to 2021, Saudi Arabia, Lebanon, and Morocco showed the fastest increases in both ASPR and ASYR, whereas Singapore, Brunei Darussalam, and Ukraine exhibited the steepest declines (Supplementary Table S1; Figures 1E,F).

### 3.4 Sex-specific pattern

In 2021, women accounted for a larger number of people with potential rehabilitation needs than men, with the gap most pronounced in older age groups, particularly among those aged 70 years and above. In some younger and middle-aged groups, men had similar or slightly higher numbers, suggesting variation in sex differences across age groups (Supplementary Table S2; Figure 2A). The distribution of YLDs showed a comparable pattern, with women carrying a greater overall disability burden, especially after age 55, and reaching a peak at 65–74 years (Supplementary Table S2; Figure 2B).

**Figure 2:**
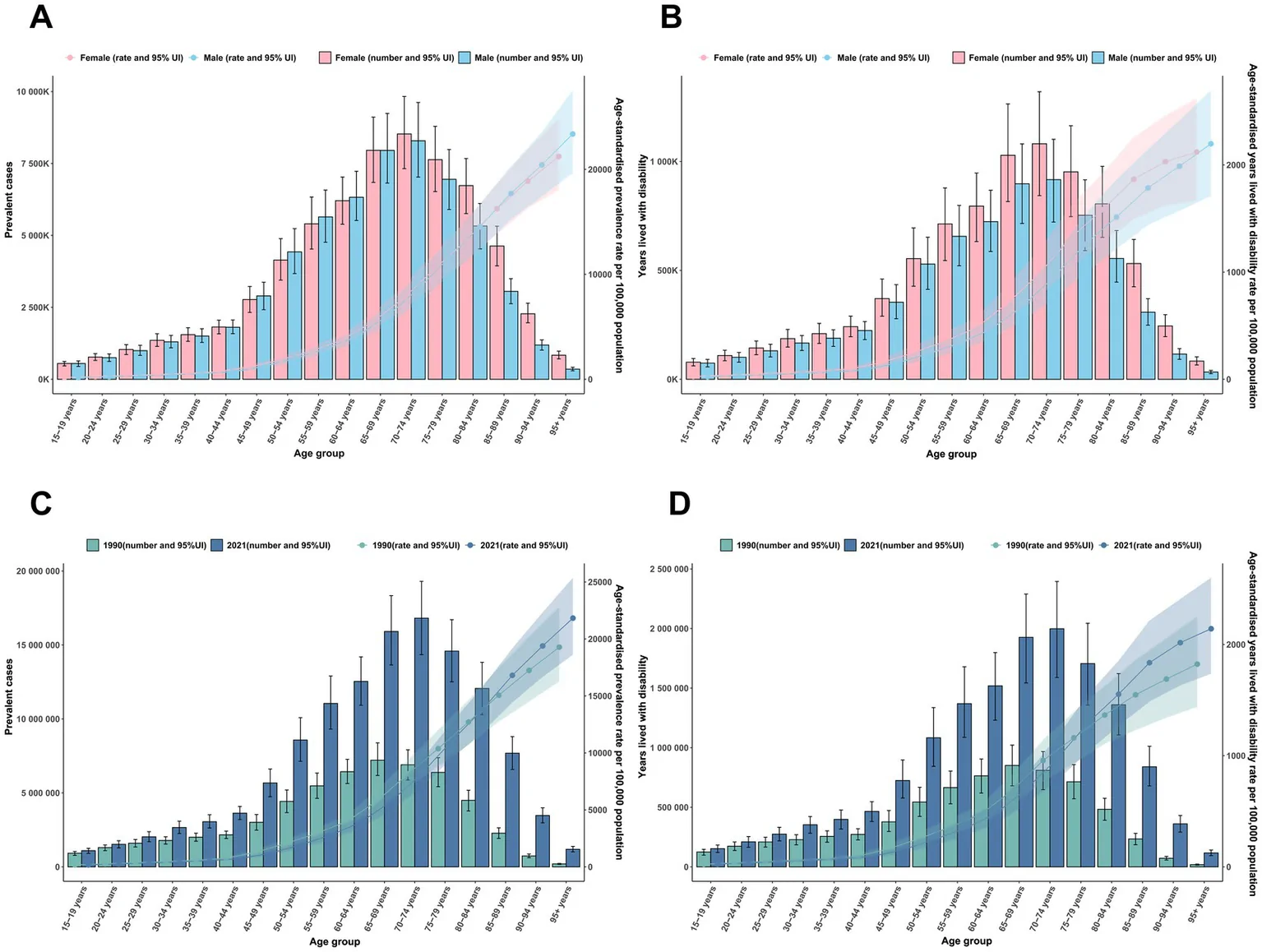
Global prevalence **(A)** and years lived with disability (YLDs) **(B)** of chronic obstructive pulmonary disease (COPD) by sex in 2021, and prevalence **(C)** and YLDs **(D)** by age in 1990 and 2021. Prevalence estimates represent population-level indicators of potential rehabilitation needs.

The age-standardized rates showed a different pattern. Among those aged 15–44 years, women had slightly higher ASPR values, but the difference was small. After age 45, male ASPRs began to exceed those of females, and the gap widened steadily, reaching a peak in the oldest age groups (Supplementary Table S2; Figure 2A). In contrast, ASYRs were higher in women across nearly all age groups, with the difference widening with age until 95 years and above, when male ASYRs slightly exceeded female values (Supplementary Table S2; Figure 2B).

### 3.5 Age-specific pattern

In 2021, both potential rehabilitation needs and YLDs generally increased with age, peaking among those aged 65–74 years at approximately 16.8 million and 2.0 million, respectively, before declining slightly at older ages. However, potential rehabilitation needs and YLDs remained high among those aged 80 years and older. Compared with 1990, increases were observed across all age groups, with the largest relative rise among the oldest age groups, where potential rehabilitation needs increased by about 4.7-fold in those aged 90–94 years and more than 6-fold in those aged 95 years and older (Supplementary Table S3; Figures 2C,D).

For ASRs, both ASPR and ASYR rose steadily with age, although patterns differed. ASPR was below 800 per 100,000 among those aged 15–44 years but rose sharply after age 45, reaching a peak of 21826.7 per 100,000 among those aged 95 years and older. Compared with 1990, ASPR declined among people aged 15–64 years, remained stable in those aged 65–74 years, and increased substantially in those aged 75 years and older (Supplementary Table S3; Figure 2C). ASYR followed a similar pattern, remaining below 100 per 100,000 for those aged 15–44 years, increasing gradually thereafter, and peaking at 2141.7 per 100,000 among those aged 95 years and older. Compared with 1990, ASYR declined among those aged 15–59 years, remained stable among those aged 60–74 years, and rose sharply at ages 75 years and above, with the greatest increases observed in the oldest groups (Supplementary Table S3; Figure 2D).

### 3.6 Projections

According to the BAPC model, the number of people with potential rehabilitation needs is projected to increase from 123.1 million in 2022 to 150.2 million in 2050. Similarly, YLDs are projected to rise from 14.8 million to 18.6 million over the same period. In contrast, both ASPR and ASYR are projected to decline, with ASPR decreasing from 2038.0 to 1940.7 per 100,000 population and ASYR from 245.0 to 239.9 per 100,000 population by 2050 (Supplementary Table S4; Figure 3).

**Figure 3:**
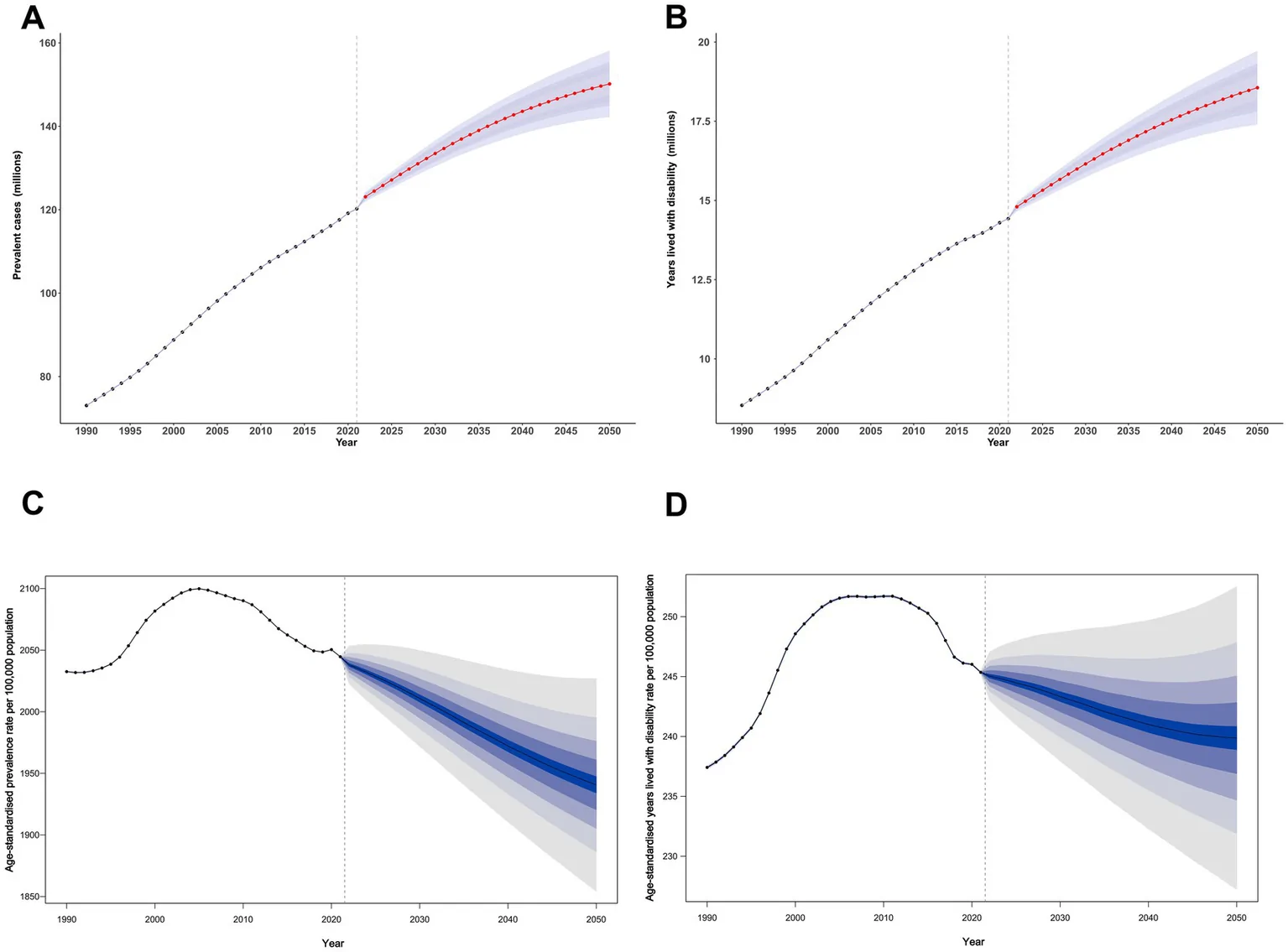
Projected numbers of prevalent cases **(A)** and years lived with disability (YLDs) **(B)**, and age-standardized rates of prevalence **(C)** and YLDs **(D)** for chronic obstructive pulmonary disease (COPD) at the global level from 2022 to 2050. Prevalence estimates represent population-level indicators of potential rehabilitation needs.

### 3.7 Decomposition analysis

Between 1990 and 2021, the number of people with potential rehabilitation needs increased by approximately 66 million, while YLDs increased by approximately 8 million. Decomposition analysis indicated that these increases were predominantly attributable to demographic factors. The increase in potential rehabilitation needs was primarily attributable to population growth (63.7%) and population aging (36.4%), whereas epidemiological change contributed minimally (−0.1%) (Supplementary Table S5; Supplementary Figure S3A). Similarly, the increase in YLDs was largely explained by population growth (62.5%) and aging (34.2%), with a relatively small contribution from epidemiological change (3.4%) (Supplementary Table S6; Supplementary Figure S3B).

At the regional level, increases in potential rehabilitation needs and YLDs in the African Region and Eastern Mediterranean Region were mainly driven by population growth, whereas population aging contributed little or negatively. In the European Region and Western Pacific Region, population aging was the principal driver, while epidemiological change contributed negatively. In the Region of the Americas, the South-East Asia Region, and the World Bank High Income group, both population growth and population aging contributed substantially to the observed increases. Overall, demographic factors, particularly population growth and population aging, were the dominant contributors to increases in potential rehabilitation needs and YLDs across regions (Supplementary Table S5, S6; Figure S3).

### 3.8 SDI-related correlations and inequality analysis

At the national level, ASPR showed no significant correlation with SDI in 2021 (*r* = −0.09, 95% CI − 0.22 to 0.04, *p* = 0.190), suggesting no statistically significant association between age-standardized potential rehabilitation needs and SDI level (Figure 4A). In contrast, ASYR was significantly and negatively correlated with SDI (*r* = −0.55, 95% CI − 0.63 to −0.44, *p* < 0.001), indicating a higher disability burden in countries with lower SDI levels (Figure 4B).

**Figure 4:**
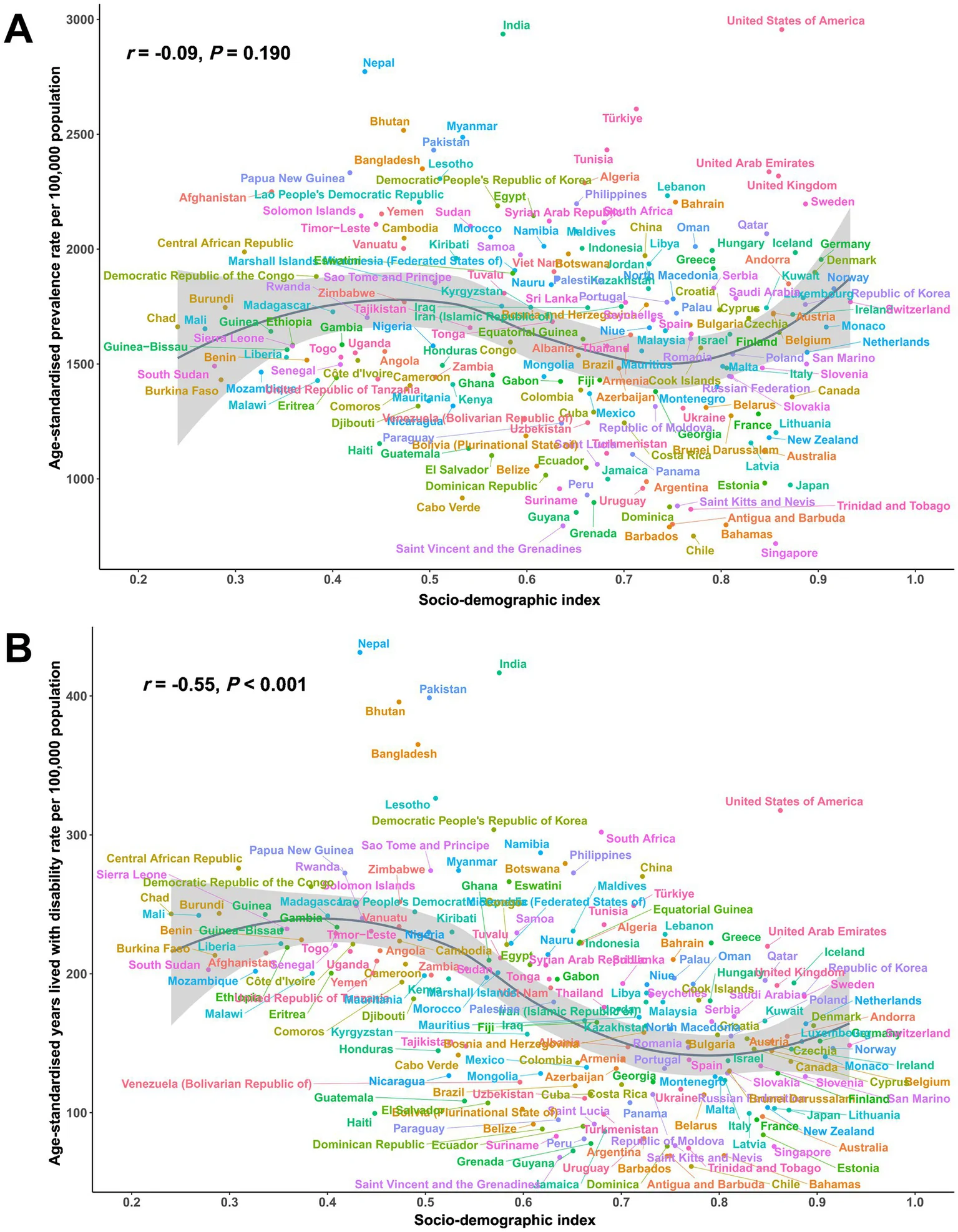
Correlations between the socio-demographic index (SDI) and age-standardized rates of prevalence **(A)** and years lived with disability (YLDs) **(B)** for chronic obstructive pulmonary disease (COPD) at the national level in 2021. Prevalence estimates represent population-level indicators of potential rehabilitation needs.

Consistent with these findings, inequality analyses revealed distinct socioeconomic patterns. SII values for ASPR remained close to zero, changing from −47.49 (95% CI − 251.66 to 156.68) in 1990 to −158.34 (95% CI − 351.27 to 34.59) in 2021, suggesting slightly higher ASPRs in lower-SDI countries, although overall disparities remained limited (Figure 5A). In contrast, inequalities in ASYR were more pronounced, with SII decreasing from −90.37 (95% CI − 113.79 to −66.96) in 1990 to −102.55 (95% CI − 126.35 to −78.75) in 2021, indicating a greater concentration of disability burden in lower-SDI settings (Figure 5B). Similar patterns were observed in CII analyses. The CII for ASPR remained near zero (−0.01 in 1990 and −0.02 in 2021), suggesting minimal relative inequality (Figure 5C), whereas ASYR CII decreased from −0.10 to −0.11, indicating a persistent concentration of disability burden among countries with lower SDI levels (Figure 5D).

**Figure 5:**
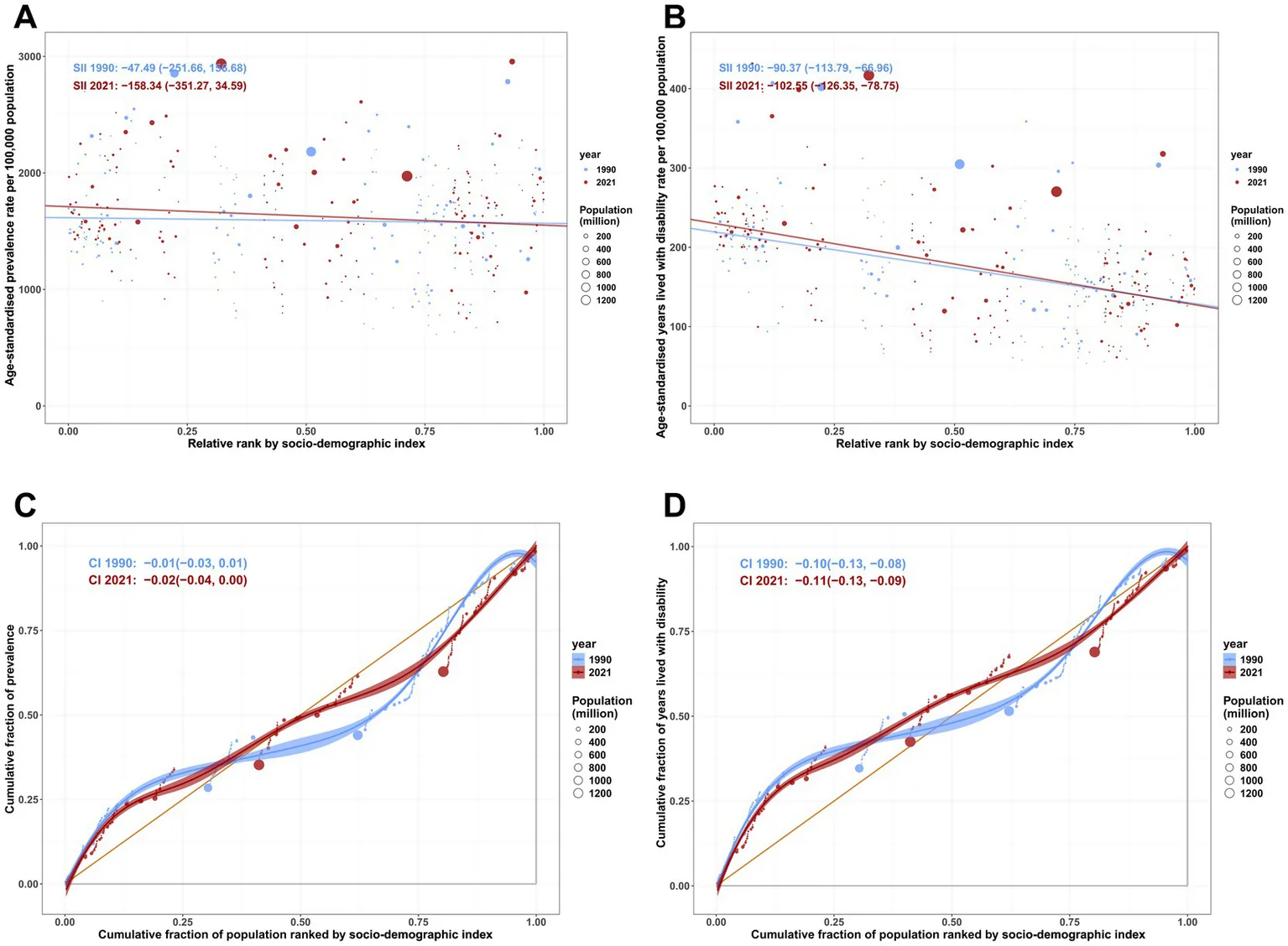
Slope index of inequality for prevalence **(A)** and years lived with disability (YLDs) **(B)**, and concentration index of inequality for prevalence **(C)** and YLDs **(D)** for chronic obstructive pulmonary disease (COPD) at the national level in 1990 and 2021. Prevalence estimates represent population-level indicators of potential rehabilitation needs.

## 4 Discussion

Our study provides a global assessment of potential rehabilitation needs among people with COPD using the WHO Rehabilitation Need Estimates framework and extends previous research that has primarily focused on prevalence, mortality, and DALYs. We described the temporal and geographical patterns of COPD-related potential rehabilitation needs and disability burden, and examined their associations with demographic, epidemiological, and socioeconomic factors. These findings provide updated evidence to inform health policy, rehabilitation planning, and priority setting for COPD management.

This study also complements traditional burden indicators, such as DALYs, by assessing potential rehabilitation needs. Compared with mortality-based measures, estimates of potential rehabilitation needs capture populations with conditions considered amenable to rehabilitation and those who may potentially benefit from rehabilitation interventions. This approach provides an additional perspective for characterizing COPD-related health loss beyond traditional mortality-based indicators.

From 1990 to 2021, potential rehabilitation needs among people with COPD and YLDs increased substantially worldwide, whereas ASPR remained relatively stable and ASYR increased slightly. These findings suggest that the growing burden was mainly attributable to demographic changes, particularly population growth and population aging, rather than major increases in age-standardized rates. Decomposition analysis further indicated that these demographic factors were the primary contributors to the observed increases. The highest numbers of potential rehabilitation needs and YLDs were observed in the South-East Asia and Western Pacific Regions, while the fastest increases occurred in the African and Eastern Mediterranean Regions. China, India, and the United States accounted for the largest national burdens. Sex-specific analyses showed higher numbers among women, whereas men had higher ASPRs in older age groups. Despite relatively stable age-standardized rates, projections indicate that the burden is likely to continue increasing through 2050. In addition, disability burden was disproportionately concentrated in lower-SDI countries, highlighting persistent socioeconomic inequalities.

Whereas previous COPD burden studies have reported declines in some age-standardized COPD burden indicators over time (5, 22), our analysis showed a different pattern. ASPR remained stable, whereas ASYR increased gradually at the global level. Although both ASPR and ASYR are projected to decline in the future, the number of people with potential rehabilitation needs and YLDs is projected to continue increasing through 2050. These findings highlight the predominant contribution of population growth and population aging to the observed increases. Other factors, including changes in disease severity, healthcare access, rehabilitation availability, and future risk factor patterns, may also influence future potential rehabilitation needs but were not directly captured in this analysis. These trends may have important implications for health and rehabilitation systems, particularly in resource-limited and rapidly aging settings. Addressing these challenges will require expanding rehabilitation capacity, improving access to rehabilitation services, and strengthening comprehensive COPD management.

The COVID-19 pandemic may have influenced future COPD rehabilitation needs. Evidence suggests that post-COVID-19 sequelae, including impaired physical and cognitive function, may increase the number of people with COPD who could benefit from rehabilitation (23, 24). These potential long-term effects should be considered in future rehabilitation planning.

Previous studies have shown that COPD prevalence and mortality are generally higher among men, although sex differences have narrowed in some high-income settings (25). In the present study, men exhibited higher ASPRs than women after the age of 45 years, which may be related to the historically higher prevalence of smoking and occupational exposures among men. In contrast, women had higher ASYRs across most age groups, which may reflect differences in exposure patterns, biological susceptibility, and disease progression (26–28). Women also accounted for higher absolute numbers of potential rehabilitation needs and YLDs, which may partly reflect their longer life expectancy and greater representation in older age groups. These findings highlight the importance of considering sex- and age-specific approaches in COPD prevention, rehabilitation planning, and long-term disease management.

The burden of COPD increased progressively with age, and both potential rehabilitation needs and YLDs were concentrated among older adults (1, 29, 30). Several factors may contribute to this pattern, including age-related declines in lung function, accumulation of comorbidities, and longer survival among individuals living with COPD. Given the substantial burden observed in older populations, rehabilitation strategies may benefit from greater emphasis on early identification, timely intervention, and continuity of care across community and long-term care settings. Such approaches may help maintain functional capacity and improve quality of life among older adults with COPD.

Previous studies have shown that poverty and low socioeconomic status are associated with an increased risk of COPD and poorer respiratory outcomes (31–33). In this study, ASPR showed no significant association with SDI, whereas ASYRs were significantly and negatively correlated with SDI. Consistently, inequality analyses indicated relatively small socioeconomic differences in ASPRs but more pronounced disparities in disability burden, which remained concentrated in lower-SDI countries. These findings suggest that although age-standardized prevalence rates showed limited variation across SDI levels, the disability burden associated with COPD is disproportionately concentrated in lower-SDI settings. This discrepancy may reflect differences in disease severity, delayed diagnosis, survival patterns, comorbidity burden, treatment access, and availability of rehabilitation resources across countries. In particular, limited healthcare access and delayed disease management in lower-SDI settings may be associated with greater disability among individuals living with COPD. Strengthening COPD prevention, improving early diagnosis and management, and expanding access to rehabilitation services may help reduce disability burden and related inequalities, particularly in lower-SDI regions.

This study has several limitations. First, the analyses were based on the WHO Rehabilitation Need Estimates, in which prevalence-based estimates were used as indicators of potential rehabilitation needs rather than direct measures of rehabilitation service utilization, referral, unmet need, or healthcare capacity gaps. Therefore, these estimates should be interpreted as potential population-level needs rather than actual rehabilitation demand. Second, data availability and quality vary across countries, particularly in low- and middle-income settings, which may introduce uncertainty into the estimates despite standardized modeling procedures. Differences in COPD diagnostic criteria and underlying epidemiological data sources may also contribute to heterogeneity and affect comparability across locations and over time. Third, the estimates do not capture important contextual factors, including rehabilitation workforce availability, healthcare infrastructure, insurance coverage, patient awareness, and barriers to service uptake, which may influence real-world rehabilitation access. Finally, the BAPC projections are subject to modeling assumptions and uncertainty and should be interpreted as plausible future scenarios rather than precise forecasts. Future COPD patterns may also be affected by changes in tobacco control policies, air pollution exposure, climate-related events, and healthcare practices, which are difficult to predict over long time horizons. Future studies should strengthen primary data collection, improve assessment of population-level rehabilitation needs and service access, and refine projection methods to enhance the accuracy and policy relevance of estimates.

## 5 Conclusion

Based on the WHO Rehabilitation Need Estimates, this study provides a comprehensive assessment of the global burden and distribution of potential rehabilitation needs and YLDs among people with COPD. From 1990 to 2021, both indicators increased substantially, and these increases were primarily attributable to population growth and population aging, with further increases projected by 2050. Despite the overall increase, substantial heterogeneity was observed across populations and regions. Women had higher numbers of potential rehabilitation needs and YLDs, whereas men had higher ASPRs in older age groups. In addition, the burden was increasingly concentrated in older populations and in countries with lower SDI, where inequality in YLDs was more pronounced. Overall, these findings highlight the growing potential rehabilitation needs among people with COPD, particularly in the context of population aging and socioeconomic inequalities. Strengthening prevention strategies, improving access to rehabilitation services, and reducing socioeconomic disparities will be important to address the future global burden of COPD.

## Data Availability

Publicly available datasets were analyzed in this study. This data can be found at: WHO Rehabilitation Need Estimates, available at: https://vizhub.healthdata.org/rehabilitation/.
