# Supplementary material for: Global trends and projections of rehabilitation needs in chronic obstructive pulmonary disease, 1990–2050: an analysis using WHO rehabilitation need estimates
Source: Front Public Health. 2026 Sep 14;14:1921263. doi: 10.3389/fpubh.2026.1921263 (PMC13637670; doi:10.3389/fpubh.2026.1921263)
Supplement: Supplementary file 1 [file Data_Sheet_1.DOCX]

Supplementary Material

1 **Supplementary Methods**
1.1 **Calculation of ASR and EAPC**

We calculated age-standardised rates (ASRs) of prevalence and years lived with disability (YLDs) for chronic obstructive pulmonary disease (COPD) to allow comparisons across countries and regions over time. ASR was computed using the GBD 2021 standard population, with the formula:

$$\text{ASR}\text{=}\frac{\sum_{\text{i}\text{=1}}^{\text{k}} \text{a}_{\text{i}}\text{w}_{\text{i}}}{\sum_{\text{i}\text{=1}}^{\text{k}} \text{w}_{\text{i}}}\text{,}$$

where *a*_i_ is the age-specific rate for the *i*-th age group, *w*_i_ represents the population weight for that age group in the GBD reference population, and *k* is the number of age groups. All estimates were derived from 1,000 posterior draws provided by GBD, with 95% uncertainty intervals (UIs) defined by the 2.5th and 97.5th percentiles.(1)

Temporal trends in age-standardised prevalence rate (ASPR) and age-standardised YLD rate (ASYR) were quantified using the estimated annual percentage change (EAPC). For each location, we fitted a log-linear regression:

$$\ln\text{(}\text{AS}\text{R}_{\text{t}}\text{)=}\text{α}\text{+}\text{βt}\text{+}\text{ϵ}\text{,}$$

where *t* represents calendar year (1990–2021) and *β* is the slope. EAPC and its 95% confidence interval (CI) were calculated as:

$$\text{EAPC}\text{=100×(}\text{e}^{\text{β}}\text{-}\text{1),}$$

$$\text{EAP}\text{C}_{\text{l}\text{ower}\text{/}\text{upper}}\text{=100×}\left( \text{e}^{\text{β}\text{±1.96×}\text{SE}}\text{-}\text{1} \right)\text{,}$$

where SE is the standard error of β. A trend was considered significant if the 95% CI excluded zero. Positive EAPC values indicated an increasing trend, negative values a decreasing trend, and values with CIs including zero indicated no significant change.(2)

1.2 **Bayesian age–period–cohort model projections**

Future trends in the numbers and ASRs of rehabilitation needs (prevalence) and YLDs attributable to COPD from 2022 to 2050 were projected using Bayesian age–period–cohort (BAPC) models. Data inputs included prevalence and YLDs attributable to COPD, age- and year-specific population counts for 1990–2021, projected counts for 2022–2050, and age-standardisation weights. Demographic and outcome data were harmonised and integrated before model fitting. Bayesian inference was implemented via integrated nested Laplace approximations (INLA)(3) in R (version 4.4.2) to jointly estimate age, period, and cohort effects, producing projections for both absolute numbers and ASRs.

**In the BAPC model, age and cohort effects were modeled using second-order random walk (RW2) priors, while the period effect was modeled using a first-order random walk (RW1) prior to achieve temporal smoothing. An independent and identically distributed (iid) random effect was incorporated to account for potential overdispersion. Precision hyperparameters for the age, period, and cohort effects were assigned log-Gamma prior distributions with parameters (1, 0.00005), whereas the precision parameter for the overdispersion term was assigned a log-Gamma prior distribution with parameters (1, 0.005).**

**Posterior inference was performed using the INLA framework. Projected estimates and corresponding uncertainty intervals were derived from the posterior distributions of the fitted Bayesian APC models. Specifically, uncertainty intervals were calculated based on posterior standard deviations under the assumption of approximate normality of posterior distributions. These uncertainty intervals represent uncertainty arising from the BAPC model itself and do not additionally incorporate uncertainty associated with the original WHO Rehabilitation Need Estimates.**

1.3 **Decomposition analysis**

We decomposed the total change in COPD rehabilitation needs (prevalence) and YLDs between 1990 and 2021 into three components: population ageing, referring to shifts in the age structure towards older age groups; population growth, representing increases in overall population size; and epidemiological change, measured through ASPR and ASYR, reflecting variation attributable to disease epidemiology, medical care, and other non-demographic determinants.(4) Following the decomposition identity:

$$\text{Δ}\text{Y}\text{ }\text{=}\text{A}\text{+}\text{P}\text{+}\text{M}\text{+}\text{Ma}\text{+}\text{Mp}\text{+}\text{Mm}\text{+}\text{I}_{\text{am}}\text{+}\text{I}_{\text{pm}}\text{+}\text{I}_{\text{pa}}\text{+}\text{I}_{\text{pam}}$$

where A, P, M denote the main effects of population ageing, population growth, and epidemiological change, respectively; M_a_, M_p_, M_m_ are their individual main effects; I_am_, I_pm_, I_pa_ are two-way interaction terms; and I_pam_ is the three-way interaction term. Interaction terms were proportionally redistributed to the main effects so that their sum equalled the observed total change. Contributions were expressed as absolute numbers and percentages relative to the 1990 baseline.(5)

1.4 **Socio-demographic index associations**

**The socio-demographic index (SDI), a composite measure of education, fertility, and income, was assigned to each location-year on a 0.0–1.0 scale. Countries were ranked by SDI using population weights and classified into quintiles: low-SDI (0–0.466), low-middle-SDI (0.466–0.619), middle-SDI (0.619–0.712), high-middle-SDI (0.712–0.810), and high-SDI (0.810–1.000) following GBD 2021 definitions.**(6) We examined the associations between ASRs of rehabilitation needs and YLDs and the SDI at the national level using Spearman’s rank correlation coefficient (*r*), with locally weighted scatterplot smoothing (LOESS) regression applied to fit the trends.

1.5 **Health inequality metrics**

**Health inequalities were quantified using two complementary measures: the slope index of inequality (SII) for absolute inequality and the concentration index of inequality (CII) for relative inequality.**

**Absolute inequality was assessed using the SII, estimated from a population-weighted regression of ASPR or ASYR against relative SDI rank (0**–**1). SII values indic**ate the predicted absolute difference **between the highest and lowest SDI levels, with positive values denoting concentration in higher-SDI countries and negative values in lower-SDI countries.**

**Relative inequality was evaluated using the CII, the area between the Lorenz curve, which plots cumulative ASPR or ASYR against the cumulative population ranked by SDI, and the 45° line of equality. CII** values range from −1 (concentration in lower-SDI) to +1 (concentration in higher-SDI), with values near zero indicating more equitable distribution.(7,8)

**References**

1.	Yang K, Yang X, Jin C, Ding S, Liu T, Ma B, Sun H, Zhang J, Li Y. Global burden of type 1 diabetes in adults aged 65 years and older, 1990-2019: population based study. *BMJ* (2024) 385:e078432. doi: 10.1136/bmj-2023-078432

2.	Sun P, Yu C, Yin L, Chen Y, Sun Z, Zhang T, Shuai P, Zeng K, Yao X, Chen J, et al. Global, regional, and national burden of female cancers in women of child-bearing age, 1990–2021: analysis of data from the global burden of disease study 2021. *Eclinicalmedicine* (2024) 74:102713. doi: 10.1016/j.eclinm.2024.102713

3.	Riebler A, Held L. Projecting the future burden of cancer: Bayesian age–period–cohort analysis with integrated nested Laplace approximations. *Biometrical J* (2017) 59:531–549. doi: 10.1002/bimj.201500263

4.	He R, Jiang W, Wang C, Li X, Zhou W. Global burden of pancreatic cancer attributable to metabolic risks from 1990 to 2019, with projections of mortality to 2030. *BMC Public Health* (2024) 24:456. doi: 10.1186/s12889-024-17875-6

5.	Zhang J, Pan L, Guo Q, Lai Y, Liu T, Wang H, Xie X, Li J, He L, Liu B, et al. The impact of global, regional, and national population ageing on disability-adjusted life years and deaths associated with diabetes during 1990–2019: A global decomposition analysis. *Diabetes & Metabolic Syndrome: Clinical Research & Reviews* (2023) 17:102791. doi: 10.1016/j.dsx.2023.102791

6.	Guan S-Y, Zheng J-X, Feng X-Y, Zhang S-X, Xu S-Z, Wang P, Pan H-F. Global burden due to modifiable risk factors for autoimmune diseases, 1990–2021: Temporal trends and socio-demographic inequalities. *Autoimmunity Reviews* (2024) 23:103674. doi: 10.1016/j.autrev.2024.103674

7.	Jiang C-Y, Han K, Yang F, Yin S-Y, Zhang L, Liang B-Y, Wang T-B, Jiang T, Chen Y-R, Shi T-Y, et al. Global, regional, and national prevalence of hearing loss from 1990 to 2019: A trend and health inequality analyses based on the Global Burden of Disease Study 2019. *Ageing Research Reviews* (2023) 92:102124. doi: 10.1016/j.arr.2023.102124

8.	Lu M, Li D, Hu Y, Zhang L, Li Y, Zhang Z, Li C. Persistence of severe global inequalities in the burden of hypertension heart disease from 1990 to 2019: findings from the global burden of disease study 2019. *BMC Public Health* (2024) 24:110.

**2. Supplementary Tables**

**Table S1.** Burden of prevalence and YLDs for COPD in 1990 and 2021, and trends (1990–2021) at national and territorial levels.

|  | **Prevalence** | | | | | **YLDs** | | | | |
| --- | --- | --- | --- | --- | --- | --- | --- | --- | --- | --- |
|  | **1990** | | **2021** | |  | **1990** | | **2021** | |  |
| **Location** | **Number**  **(95% UI)** | **ASR**  **per 100 000**  **(95% UI)** | **Number**  **(95% UI)** | **ASR per 100 000**  **(95% UI)** | **EAPC (95% CI)** | **Number**  **(95% UI)** | **ASR**  **per 100 000**  **(95% UI)** | **Number**  **(95% UI)** | **ASR per 100 000**  **(95% UI)** | **EAPC (95% CI)** |
| Afghanistan | 100060(83404,117023) | 2079.56(1726.38,2446.60) | 183300(153606,215267) | 2249.77(1863.35,2674.06) | 0.16(0.13,0.18) | 9750(7395,12415) | 201.17(152.85,255.68) | 17822(13394,22917) | 214.95(164.65,273.16) | 0.13(0.10,0.15) |
| Albania | 23631(18925,28992) | 1581.04(1268.30,1934.09) | 49081(39446,60433) | 1626.83(1306.52,2001.46) | 0.14(0.05,0.23) | 2197(1597,2910) | 144.59(106.98,189.24) | 4425(3315,5772) | 147.36(109.55,193.20) | 0.12(0.03,0.21) |
| Algeria | 176076(145633,210342) | 1903.90(1559.80,2300.44) | 575765(474605,684119) | 2288.52(1886.26,2718.10) | 0.60(0.55,0.66) | 17353(12939,22546) | 185.17(139.19,239.52) | 56360(42511,72074) | 222.22(168.45,283.17) | 0.59(0.53,0.64) |
| Andorra | 805(656,963) | 1996.59(1627.85,2386.04) | 2008(1628,2435) | 1849.16(1500.26,2240.64) | -0.29(-0.32,-0.26) | 68(50,89) | 167.70(124.95,217.70) | 167(127,218) | 154.98(116.29,202.61) | -0.28(-0.31,-0.25) |
| Angola | 51682(41977,62404) | 1643.54(1328.89,1989.96) | 144463(116412,175984) | 1554.68(1249.57,1895.46) | -0.30(-0.35,-0.25) | 7332(5346,9568) | 229.27(170.34,296.44) | 20493(14814,26907) | 216.61(160.20,280.78) | -0.29(-0.34,-0.25) |
| Antigua and Barbuda | 242(186,312) | 613.58(474.82,789.17) | 586(449,743) | 802.69(613.96,1020.10) | 0.87(0.81,0.92) | 21(14,30) | 53.68(36.55,74.73) | 51(35,69) | 68.92(47.76,94.41) | 0.82(0.77,0.87) |
| Argentina | 223414(175041,279050) | 989.20(774.82,1236.02) | 392927(314608,482810) | 988.10(792.25,1213.07) | 0.06(-0.04,0.15) | 18467(13035,25059) | 81.56(57.68,110.51) | 32234(23637,42996) | 81.32(59.46,108.54) | 0.06(-0.03,0.15) |
| Armenia | 30138(24348,36597) | 1575.50(1272.37,1914.57) | 47035(37972,57687) | 1560.78(1260.01,1912.32) | -0.01(-0.04,0.02) | 2713(2004,3556) | 140.50(104.66,183.10) | 4150(3126,5418) | 138.16(103.71,180.90) | -0.03(-0.06,0.00) |
| Australia | 206583(167684,247765) | 1483.46(1203.97,1780.44) | 362065(296061,441816) | 1121.07(915.60,1372.10) | -0.89(-1.01,-0.77) | 17649(13075,22900) | 126.69(93.80,164.51) | 31257(23502,40302) | 97.23(72.43,126.62) | -0.87(-1.02,-0.72) |
| Austria | 139069(113948,167635) | 1694.70(1388.76,2040.54) | 218404(179649,264202) | 1721.92(1416.89,2076.92) | 0.05(-0.08,0.18) | 12203(9130,15839) | 148.96(110.72,194.17) | 19056(14403,24682) | 151.17(112.86,196.39) | 0.04(-0.08,0.16) |
| Azerbaijan | 53250(43254,64132) | 1458.75(1184.28,1762.95) | 101739(81575,124214) | 1481.82(1183.20,1813.94) | 0.02(-0.04,0.08) | 4850(3535,6359) | 131.59(97.16,171.22) | 9140(6641,11991) | 131.80(96.36,172.24) | -0.03(-0.09,0.03) |
| Bahamas | 784(602,997) | 671.36(520.35,850.36) | 2259(1777,2843) | 799.87(628.52,1005.58) | 0.58(0.52,0.64) | 70(46,99) | 58.91(40.32,81.32) | 197(138,266) | 69.16(48.68,92.96) | 0.52(0.46,0.58) |
| Bahrain | 3223(2651,3856) | 2140.99(1740.63,2566.12) | 14919(12158,18085) | 2204.80(1810.61,2641.84) | -0.02(-0.06,0.03) | 317(233,412) | 207.49(155.82,264.92) | 1440(1072,1881) | 210.30(160.66,269.46) | -0.06(-0.11,-0.01) |
| Bangladesh | 818237(678367,964616) | 2315.98(1914.31,2737.41) | 2284973(1891052,2708304) | 2349.88(1947.46,2776.88) | 0.08(0.02,0.15) | 127820(95892,163818) | 358.05(272.38,455.36) | 356538(266586,455870) | 365.07(274.81,464.18) | 0.19(0.08,0.30) |
| Barbados | 1313(1017,1684) | 632.76(491.35,812.62) | 2783(2162,3516) | 790.83(613.35,1000.37) | 0.71(0.67,0.76) | 115(79,159) | 55.83(38.18,77.25) | 240(169,322) | 68.66(47.74,93.12) | 0.67(0.63,0.71) |
| Belarus | 141834(115762,172127) | 1577.36(1286.94,1911.89) | 143051(115449,174968) | 1310.99(1058.75,1603.46) | -0.71(-0.77,-0.64) | 12364(9222,16142) | 137.63(102.41,180.14) | 12252(9005,15961) | 112.89(82.21,147.86) | -0.74(-0.81,-0.67) |
| Belgium | 184517(151192,221962) | 1738.79(1421.34,2093.00) | 280070(228236,337831) | 1717.68(1397.73,2071.96) | -0.04(-0.10,0.01) | 15408(11610,20091) | 145.68(109.01,190.80) | 23288(17637,30432) | 144.52(107.99,189.97) | -0.02(-0.08,0.03) |
| Belize | 631(492,796) | 880.47(691.63,1107.06) | 2324(1835,2877) | 1055.57(834.92,1304.00) | 0.65(0.63,0.68) | 56(38,77) | 77.60(54.27,104.69) | 205(145,276) | 91.75(65.94,122.19) | 0.61(0.59,0.63) |
| Benin | 23590(19406,28244) | 1451.13(1186.92,1749.77) | 66875(55090,80047) | 1516.96(1241.17,1831.74) | 0.14(0.08,0.19) | 3514(2577,4528) | 213.89(158.21,273.92) | 10029(7359,13003) | 224.50(166.73,288.83) | 0.15(0.11,0.20) |
| Bhutan | 4900(4097,5656) | 2697.05(2260.77,3106.35) | 10973(9118,12887) | 2517.31(2097.15,2946.30) | -0.27(-0.29,-0.25) | 780(584,984) | 425.50(323.70,530.52) | 1731(1305,2183) | 395.71(300.99,495.63) | -0.28(-0.30,-0.26) |
| Bolivia (Plurinational State of) | 26778(20941,33668) | 1148.14(897.65,1446.07) | 74802(58152,94274) | 1187.11(922.51,1493.58) | 0.10(0.06,0.13) | 2391(1656,3263) | 100.65(71.25,135.00) | 6536(4568,8771) | 102.53(72.60,136.23) | 0.06(0.03,0.09) |
| Bosnia and Herzegovina | 49090(39625,59795) | 1717.71(1387.18,2096.07) | 75116(61412,90942) | 1757.65(1436.20,2126.69) | 0.14(0.11,0.16) | 4504(3338,5912) | 155.50(115.86,203.41) | 6678(5050,8626) | 157.81(118.12,204.69) | 0.11(0.09,0.14) |
| Botswana | 8418(6873,10111) | 1940.23(1574.61,2335.48) | 22170(18124,26689) | 1979.41(1616.30,2382.85) | 0.03(-0.05,0.11) | 1223(907,1589) | 278.68(208.51,359.53) | 3156(2344,4104) | 279.40(209.24,360.45) | -0.01(-0.10,0.08) |
| Brazil | 995517(789723,1213270) | 1555.36(1230.95,1900.89) | 2709765(2148727,3319093) | 1537.63(1219.86,1880.77) | -0.08(-0.11,-0.04) | 78720(58683,101006) | 120.97(90.39,154.56) | 210646(157160,270101) | 119.40(89.35,152.76) | -0.09(-0.12,-0.06) |
| Brunei Darussalam | 1297(1044,1574) | 1743.02(1419.85,2086.88) | 2850(2262,3513) | 1274.73(1016.16,1557.58) | -1.15(-1.20,-1.10) | 137(99,180) | 180.48(136.11,231.35) | 297(215,397) | 129.87(96.18,170.36) | -1.19(-1.25,-1.14) |
| Bulgaria | 140642(114871,169853) | 1685.51(1371.40,2043.20) | 162365(129777,196565) | 1669.65(1337.60,2020.30) | -0.03(-0.06,0.00) | 12777(9624,16502) | 153.03(114.56,198.37) | 14458(10773,18652) | 150.94(111.58,196.33) | -0.03(-0.07,0.00) |
| Burkina Faso | 44824(36682,53810) | 1325.95(1080.82,1598.70) | 109595(90276,131573) | 1431.42(1175.73,1729.90) | 0.24(0.19,0.29) | 6739(4930,8795) | 196.96(145.49,255.35) | 16545(12124,21564) | 213.28(158.42,275.34) | 0.26(0.21,0.31) |
| Burundi | 30401(24722,36468) | 1598.38(1286.10,1934.74) | 72990(60204,86757) | 1745.97(1426.10,2095.73) | 0.15(0.08,0.21) | 4332(3170,5651) | 225.05(166.26,292.32) | 10340(7628,13405) | 243.46(181.89,313.86) | 0.12(0.06,0.19) |
| Cabo Verde | 1503(1207,1838) | 899.65(728.57,1094.80) | 3137(2537,3852) | 916.92(742.44,1121.22) | 0.06(-0.04,0.15) | 239(171,316) | 143.22(104.03,188.14) | 485(350,645) | 141.49(103.07,186.34) | -0.02(-0.09,0.05) |
| Cambodia | 68542(56079,81217) | 1997.55(1620.26,2388.25) | 179823(148038,215495) | 2047.70(1676.80,2457.92) | 0.05(0.02,0.09) | 7570(5651,9762) | 217.94(164.48,278.76) | 19878(14941,25584) | 223.61(168.99,286.39) | 0.07(0.04,0.10) |
| Cameroon | 49536(40412,59831) | 1351.19(1094.32,1641.91) | 152612(126522,183832) | 1406.36(1155.36,1706.93) | 0.08(0.02,0.14) | 7395(5386,9643) | 198.99(146.40,257.75) | 22782(16582,29680) | 206.93(152.07,267.77) | 0.08(0.01,0.14) |
| Canada | 309134(247654,378803) | 1329.25(1064.81,1628.75) | 703247(573788,848058) | 1356.68(1102.41,1641.45) | -0.13(-0.41,0.15) | 31839(23215,42189) | 137.05(99.82,181.44) | 70674(53266,90647) | 136.84(102.15,176.97) | -0.21(-0.50,0.09) |
| Central African Republic | 16111(13093,19358) | 1804.87(1455.31,2183.61) | 34580(28334,41314) | 1988.12(1611.75,2398.86) | 0.28(0.25,0.32) | 2261(1673,2952) | 249.62(186.53,323.39) | 4869(3594,6290) | 276.01(205.39,354.00) | 0.31(0.27,0.34) |
| Chad | 33440(27382,40163) | 1514.96(1232.07,1832.17) | 82149(67962,97512) | 1662.12(1361.29,1997.87) | 0.31(0.24,0.37) | 5009(3666,6548) | 225.16(166.06,292.82) | 12228(8962,15861) | 243.11(180.16,313.15) | 0.26(0.19,0.33) |
| Chile | 62784(48905,78279) | 880.80(689.24,1095.30) | 136943(104967,174009) | 751.24(576.14,954.16) | -0.45(-0.49,-0.41) | 5193(3673,7052) | 72.28(51.74,97.39) | 11137(7863,15162) | 61.30(43.08,83.54) | -0.49(-0.51,-0.47) |
| China | 13099838(10994407,15180820) | 2181.53(1823.33,2533.82) | 28504301(23442376,33885359) | 1971.44(1624.51,2337.42) | -0.30(-0.36,-0.25) | 1823536(1412362,2236769) | 304.71(235.67,374.69) | 3894169(2966898,4845741) | 270.10(206.45,335.80) | -0.37(-0.41,-0.33) |
| Colombia | 173440(138418,212562) | 1350.54(1085.51,1648.56) | 551529(441565,676770) | 1386.63(1112.05,1698.77) | -0.10(-0.18,-0.02) | 17247(12460,22843) | 131.93(98.08,171.89) | 54004(40159,70963) | 135.95(101.15,178.27) | -0.08(-0.16,0.00) |
| Comoros | 2248(1828,2703) | 1409.38(1140.73,1701.32) | 5188(4194,6336) | 1391.33(1123.36,1702.47) | -0.13(-0.20,-0.06) | 320(234,419) | 197.49(146.54,255.72) | 732(536,963) | 194.01(143.49,253.59) | -0.13(-0.19,-0.06) |
| Congo | 12569(10097,15275) | 1526.05(1223.71,1859.92) | 32857(26505,39899) | 1595.31(1285.11,1938.79) | 0.08(0.02,0.15) | 1782(1292,2332) | 213.37(156.60,277.63) | 4621(3366,6046) | 221.06(163.63,286.90) | 0.06(-0.00,0.13) |
| Cook Islands | 162(133,194) | 1795.87(1469.85,2163.94) | 275(220,338) | 1570.18(1258.96,1924.26) | -0.55(-0.59,-0.51) | 19(14,24) | 209.16(158.68,269.13) | 32(23,41) | 180.94(133.42,235.81) | -0.57(-0.61,-0.53) |
| Costa Rica | 14637(11402,18548) | 1140.33(892.58,1439.11) | 48840(38358,61146) | 1244.10(977.80,1554.09) | 0.21(0.16,0.26) | 1443(1018,1960) | 111.20(80.37,149.07) | 4714(3436,6188) | 120.23(87.65,157.74) | 0.18(0.13,0.23) |
| Croatia | 63927(51549,77718) | 1527.58(1231.83,1859.07) | 108132(89655,129805) | 1735.77(1434.74,2087.18) | 0.48(0.46,0.51) | 5788(4286,7548) | 137.65(101.88,179.96) | 9555(7340,12180) | 156.00(118.10,200.80) | 0.47(0.45,0.50) |
| Cuba | 76212(59870,95301) | 1035.13(815.85,1293.39) | 176285(141706,216929) | 1290.63(1038.52,1585.89) | 0.75(0.65,0.86) | 6757(4731,9087) | 91.39(64.61,122.12) | 15368(11341,20077) | 113.50(83.05,149.20) | 0.75(0.64,0.85) |
| Cyprus | 10366(8373,12586) | 1858.91(1503.01,2249.68) | 24948(20071,30359) | 1737.55(1399.02,2111.11) | -0.22(-0.25,-0.19) | 870(643,1143) | 155.27(115.38,202.93) | 2087(1545,2741) | 145.75(107.62,191.98) | -0.21(-0.23,-0.18) |
| Czechia | 136610(109905,168305) | 1438.40(1157.23,1772.35) | 250942(207853,298959) | 1699.85(1405.82,2030.61) | 0.57(0.55,0.59) | 12246(9036,15976) | 129.53(95.33,169.56) | 22268(17094,28639) | 153.30(116.08,198.41) | 0.58(0.56,0.60) |
| Côte d’Ivoire | 51653(42702,61657) | 1400.95(1144.85,1692.28) | 146948(121205,176627) | 1515.70(1237.56,1843.03) | 0.30(0.22,0.37) | 7577(5548,9861) | 201.15(149.52,259.75) | 21791(15866,28489) | 221.40(163.05,288.41) | 0.35(0.28,0.42) |
| Democratic People's Republic of Korea | 279813(236176,320115) | 2498.10(2116.87,2851.51) | 498789(412902,587335) | 2188.63(1809.92,2574.58) | -0.47(-0.52,-0.42) | 39818(30283,50190) | 358.55(274.87,449.96) | 68908(51645,88210) | 303.76(227.66,388.35) | -0.57(-0.63,-0.51) |
| Democratic Republic of the Congo | 198403(161124,239870) | 1633.92(1321.45,1984.84) | 536714(437796,645924) | 1881.04(1531.85,2270.51) | 0.44(0.41,0.46) | 27856(20372,36547) | 225.27(167.21,292.83) | 75928(55618,98870) | 262.87(195.56,339.35) | 0.49(0.46,0.52) |
| Denmark | 114152(93998,135490) | 2041.56(1683.90,2418.82) | 155017(127649,185328) | 1898.94(1561.18,2269.25) | -0.36(-0.42,-0.30) | 9555(7187,12253) | 171.80(128.70,220.55) | 13206(10015,16908) | 162.68(122.20,209.62) | -0.28(-0.33,-0.23) |
| Djibouti | 1575(1254,1917) | 1231.59(979.63,1502.78) | 7003(5638,8512) | 1316.85(1055.48,1602.75) | 0.11(-0.04,0.26) | 225(161,298) | 172.19(126.45,224.36) | 986(709,1289) | 181.97(133.13,235.82) | 0.08(-0.08,0.23) |
| Dominica | 329(256,418) | 777.04(605.98,983.12) | 508(396,640) | 878.13(685.06,1106.95) | 0.40(0.38,0.42) | 29(20,40) | 68.37(47.18,92.90) | 44(31,60) | 75.88(53.48,103.35) | 0.34(0.33,0.36) |
| Dominican Republic | 23110(17969,29019) | 815.62(636.06,1023.47) | 73129(57398,92189) | 1016.19(797.97,1280.00) | 0.77(0.74,0.79) | 2077(1379,2872) | 71.77(49.33,97.26) | 6383(4476,8614) | 88.26(62.56,118.41) | 0.73(0.70,0.75) |
| Ecuador | 37814(29038,47915) | 960.58(739.62,1214.13) | 121189(94761,152508) | 1048.67(821.58,1316.19) | 0.22(0.18,0.26) | 3366(2296,4620) | 84.19(58.99,113.42) | 10468(7398,14210) | 90.21(64.22,121.67) | 0.16(0.12,0.20) |
| Egypt | 339776(276059,409793) | 1633.27(1315.81,1981.95) | 969577(793972,1164274) | 2145.83(1752.17,2583.11) | 0.84(0.81,0.86) | 33564(24557,43985) | 159.08(118.12,206.74) | 94661(70276,122457) | 206.59(154.75,266.14) | 0.80(0.77,0.83) |
| El Salvador | 21947(17039,27898) | 995.05(775.68,1260.97) | 50930(39178,64640) | 1101.95(848.42,1394.36) | 0.27(0.23,0.30) | 2178(1528,2976) | 97.87(70.10,131.81) | 4928(3513,6646) | 106.98(76.39,143.88) | 0.22(0.19,0.26) |
| Equatorial Guinea | 2505(2035,3010) | 1706.99(1379.65,2057.68) | 6831(5444,8362) | 1608.55(1286.92,1963.05) | -0.21(-0.36,-0.06) | 352(258,456) | 236.44(174.92,304.36) | 965(692,1292) | 222.84(164.40,292.09) | -0.20(-0.34,-0.06) |
| Eritrea | 14627(11962,17472) | 1428.06(1153.81,1722.61) | 34480(28145,41850) | 1439.98(1166.18,1757.79) | -0.06(-0.13,0.02) | 2085(1526,2720) | 200.55(148.87,259.18) | 4871(3536,6402) | 200.41(148.06,261.30) | -0.08(-0.15,-0.00) |
| Estonia | 12230(9816,15192) | 868.21(695.20,1079.05) | 18210(14650,22283) | 982.05(788.73,1204.36) | 0.73(0.64,0.82) | 1049(750,1396) | 74.66(53.05,99.70) | 1545(1122,2042) | 84.05(60.07,112.32) | 0.70(0.59,0.81) |
| Eswatini | 4418(3577,5326) | 1902.93(1538.26,2295.85) | 8281(6762,9916) | 1894.95(1545.28,2274.31) | -0.09(-0.16,-0.01) | 644(474,846) | 274.16(204.03,355.93) | 1176(869,1530) | 266.33(199.20,343.96) | -0.17(-0.25,-0.09) |
| Ethiopia | 273128(226440,323684) | 1680.23(1375.01,2016.48) | 589115(485484,698355) | 1582.08(1293.75,1884.81) | -0.30(-0.36,-0.23) | 38612(29502,48745) | 232.16(176.89,293.86) | 83447(63234,106275) | 219.01(166.38,278.51) | -0.27(-0.34,-0.21) |
| Fiji | 4928(4067,5910) | 1811.12(1476.01,2201.12) | 7444(6098,9082) | 1430.19(1163.19,1752.46) | -0.82(-0.86,-0.78) | 581(431,756) | 210.73(157.78,272.93) | 864(641,1126) | 165.05(122.78,215.06) | -0.85(-0.89,-0.80) |
| Finland | 79869(67139,94023) | 1630.00(1368.52,1918.27) | 141421(115226,171518) | 1637.62(1333.64,1983.78) | -0.11(-0.17,-0.05) | 6205(4715,7948) | 126.98(96.06,163.28) | 10963(8264,14246) | 128.44(95.16,168.03) | -0.11(-0.17,-0.04) |
| France | 726516(569844,913042) | 1253.68(985.28,1572.91) | 1260309(996075,1570323) | 1282.75(1015.27,1597.06) | 0.14(0.08,0.20) | 52047(36462,70942) | 90.34(62.82,123.48) | 92042(66242,122742) | 94.95(67.21,127.88) | 0.25(0.17,0.32) |
| Gabon | 5454(4365,6693) | 1298.51(1039.97,1589.89) | 10997(8756,13580) | 1424.48(1135.74,1761.16) | 0.28(0.17,0.39) | 765(550,1002) | 180.61(131.61,235.32) | 1531(1106,2036) | 196.13(143.25,258.11) | 0.25(0.14,0.37) |
| Gambia | 4472(3668,5354) | 1459.56(1186.10,1767.75) | 13085(10799,15675) | 1584.01(1296.13,1915.42) | 0.28(0.23,0.34) | 670(489,873) | 215.77(158.74,279.13) | 1954(1437,2560) | 233.60(173.52,303.51) | 0.28(0.22,0.34) |
| Georgia | 50677(40570,62025) | 1182.53(947.70,1446.10) | 57167(45684,69544) | 1380.19(1108.95,1671.02) | 0.53(0.47,0.59) | 4538(3294,5973) | 105.78(76.84,139.41) | 5012(3706,6505) | 122.13(89.76,159.12) | 0.50(0.44,0.55) |
| Germany | 1789659(1477752,2125704) | 2031.72(1680.13,2407.95) | 2646500(2204878,3123207) | 1954.71(1629.74,2312.38) | -0.08(-0.21,0.04) | 139135(103293,180573) | 158.05(116.88,205.64) | 203870(155490,260943) | 151.55(114.07,195.37) | -0.13(-0.20,-0.06) |
| Ghana | 65679(53696,78593) | 1255.47(1017.07,1515.65) | 198812(163937,237976) | 1453.01(1186.28,1758.32) | 0.56(0.50,0.62) | 9584(7008,12431) | 181.07(134.22,232.95) | 29001(21385,37554) | 209.78(156.43,269.74) | 0.60(0.50,0.70) |
| Greece | 204673(165735,247792) | 1964.28(1594.26,2371.89) | 324304(269260,388334) | 1916.22(1588.09,2298.12) | -0.03(-0.10,0.05) | 20991(15558,27373) | 201.85(149.32,263.48) | 37543(28464,48847) | 222.25(166.93,291.59) | 0.53(0.36,0.70) |
| Grenada | 395(306,501) | 752.22(586.47,950.97) | 703(549,881) | 897.29(701.17,1126.73) | 0.53(0.48,0.58) | 34(24,47) | 66.04(46.42,90.38) | 61(43,83) | 77.82(54.94,105.32) | 0.50(0.45,0.55) |
| Guatemala | 26990(21039,34085) | 1074.05(835.48,1363.17) | 88913(69711,111770) | 1132.46(890.03,1415.92) | 0.06(-0.00,0.12) | 2683(1858,3653) | 104.18(74.36,139.68) | 8613(6138,11597) | 108.39(78.58,144.03) | 0.01(-0.05,0.08) |
| Guinea | 36310(29651,43777) | 1443.54(1170.71,1749.70) | 75910(62435,90445) | 1642.26(1342.17,1974.79) | 0.42(0.37,0.48) | 5440(4005,7097) | 214.41(158.82,279.23) | 11366(8394,14722) | 242.74(180.80,312.51) | 0.41(0.36,0.47) |
| Guinea-Bissau | 4953(4111,5851) | 1488.81(1218.14,1786.81) | 10173(8480,12073) | 1562.32(1278.76,1886.57) | 0.17(0.08,0.26) | 740(550,960) | 219.34(164.17,283.25) | 1531(1125,1999) | 231.74(171.25,300.38) | 0.19(0.10,0.28) |
| Guyana | 2063(1576,2636) | 705.29(541.99,896.45) | 3841(2987,4836) | 854.19(663.34,1074.76) | 0.54(0.50,0.58) | 181(119,254) | 60.49(41.47,82.41) | 330(228,451) | 72.51(50.79,98.01) | 0.52(0.48,0.56) |
| Haiti | 25666(20071,32054) | 1074.44(838.36,1344.20) | 61129(48107,76362) | 1153.53(907.39,1443.41) | 0.15(0.10,0.20) | 2281(1578,3118) | 93.68(66.29,125.83) | 5407(3765,7289) | 99.53(71.02,132.18) | 0.12(0.07,0.17) |
| Honduras | 19120(14920,23943) | 1265.37(986.97,1588.06) | 66726(52289,83312) | 1494.11(1176.63,1856.93) | 0.50(0.45,0.55) | 1924(1357,2593) | 124.88(89.87,165.71) | 6567(4719,8711) | 144.64(106.10,189.46) | 0.45(0.40,0.49) |
| Hungary | 175942(143851,213272) | 1745.04(1426.77,2111.78) | 265880(222322,315009) | 1994.31(1669.40,2355.95) | 0.48(0.45,0.50) | 15737(11774,20309) | 156.74(116.59,202.86) | 23688(18367,29975) | 180.57(138.78,229.58) | 0.52(0.49,0.55) |
| Iceland | 4512(3747,5350) | 2213.75(1841.60,2620.73) | 8075(6683,9549) | 1985.16(1643.59,2347.03) | -0.47(-0.66,-0.28) | 437(330,559) | 214.92(162.45,274.82) | 783(598,997) | 193.43(146.91,247.31) | -0.53(-0.82,-0.24) |
| India | 9461366(7988121,10784494) | 2854.13(2404.64,3254.21) | 24628494(20933665,27993196) | 2936.24(2499.75,3329.43) | 0.07(0.02,0.13) | 1353861(1060203,1647436) | 402.26(316.27,489.69) | 3517682(2769181,4270141) | 416.69(329.28,504.95) | 0.13(0.02,0.25) |
| Indonesia | 1351626(1105897,1616247) | 1803.19(1456.09,2176.66) | 3324448(2698777,3980291) | 2003.48(1619.20,2400.74) | 0.30(0.28,0.32) | 151648(118145,190021) | 199.72(154.49,251.72) | 372051(288369,463950) | 221.88(171.67,277.32) | 0.31(0.29,0.32) |
| Iran (Islamic Republic of) | 274051(223311,331926) | 1384.03(1119.13,1689.22) | 968230(788190,1164325) | 1750.47(1421.57,2106.12) | 0.73(0.70,0.76) | 27801(21478,35208) | 138.46(106.53,176.51) | 97126(75048,122887) | 174.53(134.97,220.68) | 0.72(0.70,0.75) |
| Iraq | 94178(76204,114505) | 1466.79(1186.04,1787.36) | 308646(251365,374739) | 1745.32(1422.32,2122.00) | 0.58(0.54,0.63) | 9176(6719,12106) | 141.66(105.69,184.26) | 29794(21797,39242) | 166.39(123.76,216.52) | 0.55(0.51,0.60) |
| Ireland | 55346(45223,65951) | 1937.72(1584.20,2306.95) | 95295(77561,115488) | 1715.53(1396.00,2080.57) | -0.43(-0.44,-0.41) | 4658(3484,6051) | 163.28(122.22,212.14) | 8062(6096,10443) | 145.79(109.30,189.71) | -0.39(-0.40,-0.38) |
| Israel | 61011(49913,73717) | 1803.94(1477.24,2176.45) | 141568(114090,171893) | 1629.56(1319.68,1976.64) | -0.34(-0.36,-0.31) | 5122(3799,6694) | 151.34(112.72,197.61) | 11874(8772,15551) | 137.44(101.07,180.53) | -0.32(-0.34,-0.30) |
| Italy | 949156(755506,1165918) | 1549.94(1236.64,1903.03) | 1555679(1236499,1905335) | 1482.87(1176.77,1823.09) | -0.17(-0.21,-0.13) | 78017(58806,99626) | 127.99(96.50,163.52) | 127291(96686,161723) | 123.28(93.30,157.31) | -0.14(-0.18,-0.10) |
| Jamaica | 11725(9182,14679) | 896.65(705.99,1118.60) | 22647(18290,28058) | 999.30(810.03,1231.84) | 0.33(0.26,0.39) | 1034(722,1410) | 79.04(55.77,106.62) | 1956(1414,2592) | 86.52(62.78,114.20) | 0.28(0.21,0.35) |
| Japan | 1471447(1164223,1826392) | 1258.31(996.40,1561.58) | 2741400(2154905,3443781) | 973.52(757.89,1230.82) | -0.74(-0.82,-0.66) | 153470(113654,198861) | 130.89(97.30,169.49) | 279900(205401,364079) | 101.86(74.12,133.33) | -0.67(-0.80,-0.53) |
| Jordan | 19317(15836,23214) | 1767.11(1445.18,2133.84) | 103465(84523,124972) | 1872.12(1531.03,2256.21) | 0.16(0.14,0.18) | 1909(1402,2495) | 172.01(129.32,221.50) | 10060(7436,13123) | 179.63(134.76,231.26) | 0.12(0.10,0.13) |
| Kazakhstan | 149400(122093,179784) | 1647.26(1340.47,1991.84) | 222561(183605,266030) | 1828.43(1506.16,2188.37) | 0.46(0.33,0.58) | 13502(9961,17621) | 147.71(109.84,192.12) | 19897(14946,25663) | 162.41(122.53,209.18) | 0.44(0.30,0.57) |
| Kenya | 92191(74119,111778) | 1309.17(1049.38,1594.35) | 266950(215886,323506) | 1411.10(1136.89,1711.31) | 0.13(0.07,0.18) | 13190(9994,16730) | 183.30(138.95,232.92) | 37865(28660,48109) | 196.40(148.54,249.96) | 0.12(0.07,0.17) |
| Kiribati | 563(471,660) | 2041.89(1672.41,2437.93) | 1033(876,1213) | 1960.19(1628.82,2341.45) | -0.10(-0.12,-0.09) | 66(51,85) | 239.47(183.30,307.03) | 122(93,157) | 230.06(174.92,295.43) | -0.10(-0.12,-0.08) |
| Kuwait | 8311(6748,10158) | 1443.93(1179.60,1765.38) | 41451(33708,50318) | 1744.49(1415.43,2111.96) | 0.61(0.60,0.63) | 813(578,1098) | 138.64(102.66,182.53) | 4019(2947,5271) | 166.06(124.11,214.34) | 0.59(0.57,0.61) |
| Kyrgyzstan | 41960(35067,49255) | 1959.12(1629.72,2312.96) | 58742(47743,70464) | 1746.83(1417.31,2101.94) | -0.51(-0.56,-0.45) | 3835(2908,4864) | 178.05(135.78,225.23) | 5324(3921,6923) | 156.55(116.41,202.23) | -0.55(-0.60,-0.50) |
| Lao People's Democratic Republic | 34925(29088,40782) | 2288.04(1885.39,2698.51) | 75176(62403,89115) | 2204.51(1816.80,2630.21) | -0.20(-0.25,-0.16) | 3908(2984,4969) | 253.39(194.27,321.13) | 8428(6374,10817) | 244.59(185.98,312.64) | -0.19(-0.24,-0.15) |
| Latvia | 28681(22864,35238) | 1165.20(930.98,1430.69) | 31166(25145,38344) | 1156.58(932.65,1419.50) | -0.05(-0.16,0.06) | 2466(1793,3268) | 100.51(72.65,133.67) | 2658(1973,3507) | 99.49(72.62,132.41) | -0.04(-0.15,0.07) |
| Lebanon | 26865(22044,32384) | 1722.19(1410.46,2083.97) | 98053(80076,117649) | 2232.76(1826.46,2676.58) | 0.99(0.92,1.05) | 2791(2073,3623) | 177.55(132.70,229.58) | 10016(7596,12824) | 228.44(173.53,291.85) | 0.96(0.89,1.03) |
| Lesotho | 13394(10941,16056) | 2138.96(1739.29,2573.78) | 18639(15299,22081) | 2306.52(1879.92,2752.57) | 0.22(0.16,0.28) | 1964(1463,2532) | 311.93(233.36,401.25) | 2656(1995,3393) | 326.38(245.91,417.14) | 0.12(0.05,0.18) |
| Liberia | 11552(9369,14069) | 1276.12(1030.29,1560.42) | 27711(22543,33609) | 1528.88(1228.88,1869.50) | 0.51(0.47,0.55) | 1692(1235,2225) | 184.50(136.32,241.24) | 4088(2956,5391) | 221.70(161.69,290.00) | 0.52(0.48,0.56) |
| Libya | 23708(19363,28517) | 1567.08(1276.56,1891.23) | 75573(62150,90803) | 1935.76(1588.15,2331.03) | 0.74(0.72,0.77) | 2330(1718,3062) | 152.64(114.37,198.24) | 7343(5466,9499) | 186.17(140.09,239.92) | 0.70(0.68,0.73) |
| Lithuania | 43267(34934,52905) | 1368.99(1107.16,1671.15) | 47171(38443,57471) | 1201.44(977.77,1464.60) | -0.54(-0.61,-0.47) | 3719(2754,4898) | 117.88(87.15,155.22) | 4006(3008,5203) | 103.09(76.13,135.00) | -0.55(-0.63,-0.48) |
| Luxembourg | 7184(5926,8570) | 1895.32(1563.08,2262.92) | 13384(10926,16134) | 1788.17(1460.16,2155.90) | -0.23(-0.29,-0.17) | 602(450,779) | 159.05(118.63,206.19) | 1126(848,1460) | 151.21(113.17,197.18) | -0.20(-0.26,-0.13) |
| Madagascar | 62476(50747,74794) | 1505.34(1211.23,1822.01) | 165204(137234,196706) | 1727.36(1412.05,2083.46) | 0.34(0.30,0.38) | 8857(6550,11493) | 210.20(156.80,271.43) | 23583(17574,30642) | 241.73(181.46,312.53) | 0.36(0.32,0.40) |
| Malawi | 39290(31556,47740) | 1214.46(971.78,1480.25) | 91225(74402,109913) | 1427.99(1166.34,1726.41) | 0.47(0.43,0.52) | 5584(4033,7407) | 169.99(124.88,223.35) | 13013(9501,17146) | 200.28(149.36,260.70) | 0.50(0.45,0.54) |
| Malaysia | 113625(90918,138051) | 1604.22(1283.06,1945.59) | 329603(266465,398765) | 1644.93(1329.00,1993.74) | 0.05(0.03,0.07) | 12563(9198,16532) | 175.83(130.86,228.52) | 36304(26793,47394) | 179.67(133.35,233.94) | 0.05(0.03,0.06) |
| Maldives | 1527(1257,1798) | 2255.39(1829.27,2684.95) | 5440(4455,6529) | 2077.26(1697.23,2490.86) | -0.43(-0.48,-0.38) | 172(130,221) | 251.94(190.23,321.96) | 611(456,790) | 230.80(174.63,294.91) | -0.44(-0.49,-0.39) |
| Mali | 48026(39703,56634) | 1482.70(1213.71,1766.60) | 124025(103460,146641) | 1653.53(1366.24,1977.87) | 0.34(0.28,0.40) | 7162(5309,9229) | 218.45(163.02,280.89) | 18452(13626,23741) | 242.06(179.93,309.53) | 0.33(0.27,0.39) |
| Malta | 4882(4011,5884) | 1651.10(1355.13,1989.99) | 9893(7902,12160) | 1490.09(1193.56,1829.75) | -0.43(-0.48,-0.37) | 409(303,537) | 138.02(102.40,181.32) | 818(606,1083) | 124.46(91.05,165.86) | -0.43(-0.48,-0.37) |
| Marshall Islands | 257(215,302) | 1984.88(1619.57,2383.25) | 456(385,536) | 1751.99(1439.90,2107.73) | -0.41(-0.44,-0.39) | 30(23,39) | 231.93(175.44,299.73) | 53(40,68) | 200.79(150.63,259.30) | -0.47(-0.50,-0.44) |
| Mauritania | 9749(8007,11755) | 1224.11(1000.88,1482.40) | 22788(18504,27761) | 1339.76(1085.44,1636.26) | 0.23(0.14,0.31) | 1464(1068,1915) | 182.25(134.24,236.96) | 3420(2473,4478) | 198.70(145.36,257.59) | 0.22(0.14,0.31) |
| Mauritius | 8161(6678,9835) | 1508.99(1231.95,1819.96) | 19236(15847,22984) | 1556.88(1283.14,1859.18) | -0.06(-0.13,0.01) | 904(667,1181) | 165.13(123.38,213.96) | 2090(1572,2705) | 168.80(126.70,218.31) | -0.10(-0.17,-0.02) |
| Mexico | 385734(303498,483158) | 1239.46(975.48,1553.29) | 1200709(947757,1479981) | 1371.13(1085.41,1685.23) | 0.41(0.36,0.46) | 38371(29008,49527) | 120.60(91.50,155.37) | 116853(89145,149282) | 132.66(101.55,169.07) | 0.39(0.34,0.44) |
| Micronesia (Federated States of) | 776(646,908) | 2126.43(1743.00,2522.63) | 1002(844,1189) | 1907.33(1578.84,2294.63) | -0.39(-0.41,-0.37) | 91(69,117) | 249.23(189.02,318.80) | 117(90,151) | 221.94(168.88,286.30) | -0.42(-0.44,-0.39) |
| Monaco | 838(672,1025) | 1752.27(1406.66,2144.00) | 1112(890,1365) | 1660.95(1330.64,2033.48) | -0.21(-0.26,-0.16) | 70(52,93) | 148.13(108.52,197.09) | 93(69,122) | 140.21(103.00,185.61) | -0.21(-0.26,-0.16) |
| Mongolia | 11463(9358,13875) | 1442.21(1168.16,1759.98) | 23005(18573,28108) | 1445.28(1161.78,1772.10) | 0.01(-0.03,0.04) | 1039(754,1375) | 128.75(94.69,168.53) | 2074(1506,2717) | 128.23(94.61,167.09) | -0.01(-0.05,0.02) |
| Montenegro | 5450(4337,6796) | 1240.46(988.63,1543.08) | 9203(7355,11338) | 1400.79(1119.42,1724.85) | 0.51(0.47,0.55) | 496(360,662) | 112.40(82.06,149.27) | 825(607,1085) | 125.98(92.19,166.56) | 0.50(0.45,0.54) |
| Morocco | 166386(135338,200770) | 1527.38(1237.38,1847.14) | 495255(406305,595395) | 2053.08(1680.80,2469.22) | 0.96(0.93,0.98) | 16316(11982,21400) | 148.51(110.45,193.21) | 47855(36084,61667) | 197.36(149.17,253.83) | 0.92(0.90,0.95) |
| Mozambique | 63691(51637,77045) | 1319.00(1065.25,1602.00) | 139973(113876,168401) | 1464.74(1185.62,1768.00) | 0.21(0.16,0.27) | 8947(6524,11717) | 182.36(134.65,236.89) | 19610(14332,25651) | 201.82(150.11,260.09) | 0.22(0.16,0.27) |
| Myanmar | 427945(358788,491423) | 2547.20(2115.41,2949.20) | 849394(714731,993576) | 2487.24(2085.31,2903.53) | -0.14(-0.16,-0.12) | 47715(36621,60198) | 281.06(216.89,352.66) | 94414(73434,119477) | 274.36(214.00,345.62) | -0.13(-0.14,-0.11) |
| Namibia | 9688(7901,11652) | 1960.10(1593.63,2365.95) | 20879(17044,25109) | 2012.38(1643.81,2422.68) | 0.08(0.01,0.15) | 1412(1046,1822) | 282.85(211.12,362.90) | 3003(2228,3877) | 287.15(214.92,368.60) | 0.06(-0.01,0.13) |
| Nauru | 69(57,80) | 1967.84(1596.29,2350.13) | 81(68,96) | 1845.34(1507.02,2224.90) | -0.26(-0.29,-0.23) | 8(6,10) | 225.52(169.31,290.67) | 9(7,12) | 213.84(161.75,276.88) | -0.21(-0.24,-0.19) |
| Nepal | 190054(163006,213979) | 2777.69(2392.77,3123.51) | 462074(402172,521153) | 2772.73(2429.81,3109.37) | 0.04(-0.01,0.09) | 29687(23016,36601) | 430.08(337.98,525.01) | 72178(56054,88908) | 431.39(338.47,527.52) | 0.06(0.01,0.11) |
| Netherlands | 230042(191280,275073) | 1652.64(1372.75,1975.37) | 373998(304748,450958) | 1551.16(1258.59,1877.17) | 0.08(-0.12,0.27) | 24176(18290,30896) | 174.19(131.44,222.88) | 39225(29686,50596) | 164.03(122.57,213.54) | 0.18(-0.07,0.42) |
| New Zealand | 38302(30331,47676) | 1373.64(1088.17,1708.45) | 70719(55036,89548) | 1179.52(918.48,1493.77) | -0.52(-0.62,-0.42) | 3348(2489,4360) | 120.10(89.24,156.59) | 6195(4546,8249) | 103.74(75.85,138.56) | -0.51(-0.61,-0.40) |
| Nicaragua | 12654(9811,15992) | 1103.24(859.78,1394.54) | 45124(35484,56079) | 1317.57(1041.48,1631.39) | 0.57(0.54,0.60) | 1259(877,1726) | 107.54(77.45,144.52) | 4384(3166,5860) | 126.69(93.05,167.37) | 0.54(0.50,0.57) |
| Niger | 35671(29325,42679) | 1482.30(1202.61,1797.87) | 118881(97746,141089) | 1729.12(1407.34,2076.84) | 0.58(0.51,0.65) | 5361(3930,7022) | 219.51(162.38,285.47) | 17999(13125,23362) | 257.68(189.94,332.12) | 0.62(0.54,0.69) |
| Nigeria | 492299(400614,589548) | 1394.90(1128.75,1681.16) | 1195939(976195,1434938) | 1579.05(1284.05,1903.39) | 0.43(0.39,0.47) | 72097(55230,90793) | 201.57(154.09,254.96) | 178221(135437,225432) | 230.01(174.68,291.19) | 0.47(0.42,0.51) |
| Niue | 30(25,36) | 1892.66(1557.30,2270.29) | 24(20,30) | 1658.08(1346.65,2032.51) | -0.51(-0.54,-0.49) | 4(3,5) | 223.27(169.44,288.08) | 3(2,4) | 192.24(143.80,250.46) | -0.57(-0.59,-0.54) |
| North Macedonia | 21630(17288,26456) | 1654.79(1323.36,2025.67) | 39456(31785,48472) | 1783.34(1439.03,2187.19) | 0.25(0.19,0.31) | 1978(1449,2595) | 149.98(110.54,195.91) | 3556(2659,4637) | 160.30(119.70,209.11) | 0.23(0.17,0.29) |
| Norway | 77157(62465,94015) | 1640.51(1328.88,1995.21) | 127791(103348,154214) | 1827.91(1479.72,2203.05) | 0.38(0.25,0.50) | 6030(4565,7780) | 129.35(97.80,166.71) | 10146(7744,12943) | 146.33(111.43,186.82) | 0.42(0.25,0.59) |
| Oman | 10717(8750,12871) | 1821.53(1480.64,2193.94) | 33694(27578,41052) | 2011.27(1645.42,2438.05) | 0.27(0.22,0.31) | 1041(761,1371) | 174.76(131.40,226.12) | 3270(2399,4352) | 192.61(145.60,250.20) | 0.27(0.22,0.31) |
| Pakistan | 1011284(829404,1193016) | 2472.76(2027.75,2917.22) | 2147137(1748864,2549958) | 2431.09(1986.07,2876.30) | 0.00(-0.05,0.06) | 168625(127523,212311) | 407.00(309.10,511.04) | 358691(270474,452123) | 398.66(302.05,500.56) | 0.09(-0.02,0.20) |
| Palau | 139(115,166) | 1908.64(1549.43,2295.94) | 255(212,306) | 1721.41(1418.13,2067.94) | -0.41(-0.44,-0.38) | 16(12,21) | 222.81(168.40,285.24) | 29(22,38) | 196.82(148.66,253.18) | -0.47(-0.50,-0.45) |
| Palestine | 11414(9369,13732) | 1704.12(1397.19,2058.50) | 35795(29385,43206) | 1868.86(1536.94,2256.02) | 0.29(0.26,0.32) | 1123(829,1462) | 166.25(124.76,214.65) | 3483(2569,4562) | 179.70(135.25,231.69) | 0.26(0.23,0.29) |
| Panama | 10583(8170,13549) | 975.83(756.63,1246.56) | 35349(28054,44027) | 1106.89(879.46,1376.72) | 0.26(0.20,0.32) | 1047(738,1434) | 95.41(68.82,128.78) | 3419(2497,4554) | 107.20(78.34,142.48) | 0.23(0.16,0.29) |
| Papua New Guinea | 33064(28525,37641) | 2445.88(2079.41,2802.46) | 90462(77758,103378) | 2332.72(1981.86,2684.29) | -0.16(-0.17,-0.15) | 3907(3037,4909) | 286.94(225.00,357.12) | 10667(8295,13450) | 272.51(214.84,339.39) | -0.17(-0.18,-0.16) |
| Paraguay | 18614(14408,23513) | 1131.57(877.60,1430.11) | 51802(40709,65222) | 1242.56(976.74,1564.55) | 0.27(0.25,0.30) | 1464(1004,2037) | 87.83(61.46,120.39) | 3985(2796,5444) | 94.87(67.41,128.37) | 0.22(0.20,0.25) |
| Peru | 76112(59141,96859) | 850.47(664.49,1077.09) | 225417(173980,287870) | 929.92(719.32,1183.36) | 0.42(0.29,0.56) | 6801(4640,9452) | 74.85(52.52,101.84) | 19723(13767,26829) | 81.14(57.09,109.67) | 0.39(0.26,0.53) |
| Philippines | 533667(440850,628502) | 2395.99(1965.99,2835.19) | 1313188(1083120,1551714) | 2197.88(1802.93,2608.59) | -0.39(-0.42,-0.36) | 66856(51928,82802) | 295.79(228.68,368.34) | 164860(127492,204585) | 272.67(210.04,340.23) | -0.28(-0.34,-0.21) |
| Poland | 474529(387035,571486) | 1555.71(1267.98,1874.55) | 758811(617647,916782) | 1542.72(1253.10,1864.80) | -0.06(-0.11,-0.02) | 47537(36353,60483) | 155.75(119.08,198.22) | 75440(57603,96327) | 155.20(118.28,198.04) | -0.03(-0.06,-0.00) |
| Portugal | 195562(164552,228312) | 2064.12(1736.24,2408.81) | 304659(250402,360553) | 1769.08(1448.88,2099.95) | -0.51(-0.59,-0.43) | 15169(11544,19538) | 160.23(121.37,206.34) | 22545(16878,29367) | 131.85(96.78,173.45) | -0.77(-0.85,-0.70) |
| Qatar | 2434(2030,2892) | 1848.50(1515.38,2234.29) | 20201(16547,24402) | 2067.17(1695.79,2470.02) | 0.32(0.30,0.34) | 236(168,318) | 176.44(131.30,229.79) | 1943(1390,2585) | 195.25(147.75,250.09) | 0.28(0.26,0.31) |
| Republic of Korea | 336461(270768,407844) | 1632.55(1320.24,1969.22) | 1148391(964626,1343196) | 1759.16(1471.81,2064.89) | 0.39(0.12,0.65) | 35208(25759,45936) | 166.99(125.13,214.27) | 120686(93203,151242) | 185.05(141.76,233.21) | 0.50(0.22,0.78) |
| Republic of Moldova | 48404(39694,58154) | 1583.96(1294.00,1909.01) | 53726(43050,66290) | 1315.32(1054.82,1619.88) | -0.77(-0.83,-0.71) | 4217(3135,5467) | 137.57(102.27,178.85) | 4600(3378,6073) | 113.13(82.54,149.96) | -0.80(-0.86,-0.74) |
| Romania | 329864(267043,400727) | 1720.70(1391.43,2093.01) | 412811(338105,498640) | 1610.64(1316.83,1944.83) | -0.25(-0.28,-0.22) | 30082(22347,39361) | 156.50(116.38,204.84) | 37108(27933,47874) | 147.03(109.34,191.18) | -0.23(-0.26,-0.20) |
| Russian Federation | 1902787(1539927,2289731) | 1541.81(1248.86,1857.08) | 2340903(1906820,2815508) | 1446.95(1177.69,1738.59) | -0.30(-0.34,-0.26) | 171791(132114,217432) | 139.38(107.28,176.40) | 206919(159834,261400) | 128.46(99.35,161.74) | -0.35(-0.39,-0.31) |
| Rwanda | 37624(30819,44817) | 1601.07(1295.03,1931.10) | 87758(71998,104985) | 1702.26(1390.19,2048.06) | 0.08(0.01,0.15) | 5394(3955,7019) | 226.11(167.07,293.41) | 12532(9248,16231) | 239.98(179.21,309.09) | 0.09(0.03,0.16) |
| Saint Kitts and Nevis | 182(141,230) | 690.45(537.27,875.60) | 414(319,525) | 882.46(677.74,1119.69) | 0.77(0.69,0.86) | 16(11,22) | 60.48(41.76,82.81) | 36(25,50) | 76.30(53.73,103.95) | 0.74(0.65,0.82) |
| Saint Lucia | 587(458,737) | 919.89(720.56,1151.55) | 1783(1406,2202) | 1063.71(838.89,1313.59) | 0.46(0.41,0.51) | 52(36,71) | 80.20(56.97,108.31) | 154(109,204) | 91.79(64.99,122.19) | 0.43(0.38,0.48) |
| Saint Vincent and the Grenadines | 346(265,443) | 662.76(509.20,844.39) | 782(613,983) | 795.58(623.41,1000.23) | 0.63(0.60,0.65) | 30(20,42) | 57.89(39.36,79.23) | 67(47,90) | 67.87(47.79,91.89) | 0.55(0.53,0.58) |
| Samoa | 1381(1159,1617) | 2199.49(1821.80,2603.57) | 2038(1692,2429) | 1975.26(1626.76,2365.93) | -0.40(-0.42,-0.37) | 163(123,208) | 258.32(196.63,328.79) | 238(180,307) | 229.51(173.66,294.45) | -0.43(-0.45,-0.40) |
| San Marino | 410(329,501) | 1667.39(1340.75,2036.56) | 804(643,988) | 1500.16(1198.91,1842.71) | -0.37(-0.39,-0.35) | 34(25,45) | 139.79(102.18,185.52) | 66(49,88) | 126.06(92.02,168.30) | -0.36(-0.38,-0.34) |
| Sao Tome and Principe | 773(637,925) | 1576.72(1293.76,1894.03) | 1679(1404,1980) | 1852.85(1530.32,2210.69) | 0.50(0.46,0.53) | 116(85,151) | 235.50(173.72,305.47) | 251(186,324) | 274.29(203.75,353.59) | 0.47(0.43,0.51) |
| Saudi Arabia | 64259(51301,79166) | 1315.71(1053.62,1617.23) | 281185(229331,342496) | 1785.72(1452.28,2173.83) | 1.05(1.02,1.09) | 6254(4430,8462) | 125.54(92.16,165.89) | 27298(19911,36170) | 169.29(126.16,220.67) | 1.04(1.01,1.07) |
| Senegal | 36311(29931,43518) | 1383.43(1133.36,1668.33) | 93230(76513,112266) | 1497.98(1222.32,1815.54) | 0.29(0.22,0.35) | 5401(3914,7042) | 202.97(148.85,262.98) | 13883(10140,18086) | 220.35(162.49,285.47) | 0.30(0.23,0.37) |
| Serbia | 124908(102123,151935) | 1624.78(1325.80,1977.99) | 208341(172223,250579) | 1830.39(1511.13,2198.58) | 0.44(0.40,0.48) | 11460(8600,14963) | 147.89(110.98,193.37) | 18630(14270,23876) | 165.60(125.42,213.08) | 0.42(0.38,0.46) |
| Seychelles | 621(501,757) | 1496.20(1209.40,1819.63) | 1366(1096,1656) | 1692.00(1356.24,2054.72) | 0.27(0.22,0.32) | 69(51,90) | 165.43(122.58,215.00) | 150(112,197) | 184.38(137.16,241.10) | 0.22(0.18,0.27) |
| Sierra Leone | 23551(19355,28388) | 1439.61(1175.34,1745.55) | 50334(41727,60016) | 1578.68(1300.25,1899.08) | 0.27(0.21,0.33) | 3490(2542,4568) | 211.16(155.17,274.62) | 7511(5518,9751) | 232.33(172.78,299.58) | 0.29(0.23,0.35) |
| Singapore | 17304(13594,21634) | 1128.59(893.19,1400.55) | 43070(33876,53829) | 718.41(564.13,898.25) | -1.61(-1.70,-1.52) | 1834(1321,2478) | 118.27(87.09,156.93) | 4542(3257,6149) | 75.67(54.23,102.35) | -1.60(-1.69,-1.51) |
| Slovakia | 54652(44059,67733) | 1309.68(1055.71,1617.33) | 93951(76625,114721) | 1444.83(1175.79,1765.42) | 0.37(0.35,0.38) | 4914(3605,6470) | 117.78(86.38,155.21) | 8393(6314,11008) | 130.23(96.95,171.63) | 0.38(0.36,0.40) |
| Slovenia | 25429(20414,31204) | 1471.62(1182.47,1805.44) | 46086(37447,56221) | 1483.04(1199.77,1811.81) | 0.02(0.01,0.04) | 2278(1684,3005) | 132.06(97.39,174.28) | 4074(3060,5266) | 133.68(98.91,174.47) | 0.03(0.01,0.05) |
| Solomon Islands | 2364(2015,2734) | 2316.94(1932.27,2721.20) | 5588(4757,6484) | 2144.86(1790.74,2528.87) | -0.26(-0.27,-0.25) | 278(213,352) | 268.48(206.60,338.67) | 657(502,838) | 249.12(191.46,317.24) | -0.25(-0.26,-0.24) |
| Somalia | 36917(30227,43822) | 1692.74(1366.86,2042.30) | 108504(90140,127184) | 1965.00(1605.62,2348.35) | 0.39(0.32,0.45) | 5269(3887,6877) | 237.30(176.70,308.14) | 15563(11590,20122) | 276.99(207.88,355.98) | 0.41(0.35,0.47) |
| South Africa | 330739(273399,391895) | 2114.14(1739.74,2512.56) | 701794(578717,831946) | 2115.97(1743.88,2509.11) | -0.07(-0.14,0.00) | 48318(37123,60549) | 306.19(234.85,384.26) | 100676(77128,126613) | 302.00(231.42,379.70) | -0.12(-0.21,-0.04) |
| South Sudan | 28351(22784,34398) | 1364.37(1095.62,1660.33) | 47694(38573,57752) | 1490.86(1202.60,1810.56) | 0.16(0.09,0.23) | 3920(2876,5136) | 185.46(137.80,240.60) | 6655(4869,8727) | 202.98(151.01,263.22) | 0.18(0.11,0.25) |
| Spain | 649064(530447,784551) | 1721.15(1407.92,2075.81) | 1133871(920751,1375737) | 1630.98(1326.05,1976.22) | -0.26(-0.30,-0.22) | 54581(40047,70868) | 144.99(105.87,188.79) | 95174(70908,123783) | 138.06(101.61,181.42) | -0.23(-0.27,-0.19) |
| Sri Lanka | 132509(107070,161118) | 1660.94(1336.28,2027.05) | 325477(262916,395442) | 1768.09(1427.68,2149.09) | 0.12(0.10,0.14) | 14780(10918,19237) | 183.44(136.77,236.60) | 35662(26655,45966) | 192.96(144.09,248.95) | 0.09(0.07,0.11) |
| Sudan | 143325(118033,169869) | 2022.44(1651.89,2419.78) | 322805(265769,384953) | 2099.62(1715.74,2523.04) | 0.01(-0.04,0.05) | 14091(10583,18244) | 196.72(149.13,253.14) | 31723(23565,41385) | 203.60(153.26,262.78) | -0.00(-0.05,0.04) |
| Suriname | 1587(1247,2004) | 841.29(663.73,1058.56) | 4237(3343,5339) | 957.27(755.55,1206.20) | 0.37(0.32,0.42) | 142(96,195) | 74.07(51.39,100.54) | 368(263,499) | 83.03(59.43,112.35) | 0.33(0.28,0.38) |
| Sweden | 235965(197069,277267) | 2279.41(1904.75,2670.00) | 331444(273705,391351) | 2196.69(1810.08,2597.39) | -0.11(-0.22,0.00) | 19590(15082,24782) | 189.90(145.34,240.74) | 27575(21321,34657) | 183.72(140.72,231.97) | -0.13(-0.30,0.04) |
| Switzerland | 140025(114708,168184) | 1921.89(1576.63,2303.70) | 225376(186037,268270) | 1770.78(1463.86,2109.18) | -0.38(-0.49,-0.27) | 11612(8720,15137) | 160.21(119.73,208.90) | 18683(14153,23998) | 148.47(111.27,191.99) | -0.36(-0.46,-0.25) |
| Syrian Arab Republic | 72103(59876,85699) | 1731.35(1426.35,2078.14) | 191264(158353,228110) | 2122.28(1755.54,2526.63) | 0.65(0.62,0.68) | 7107(5277,9236) | 168.67(127.06,217.19) | 18727(14177,23988) | 206.20(156.36,263.57) | 0.65(0.62,0.68) |
| Tajikistan | 34460(28392,40850) | 1665.75(1359.72,1993.80) | 70214(57507,84221) | 1657.93(1343.60,2004.78) | -0.04(-0.06,-0.01) | 3148(2351,4053) | 150.47(113.18,192.73) | 6394(4732,8341) | 147.95(111.15,191.97) | -0.07(-0.10,-0.05) |
| Thailand | 536315(457149,620663) | 1962.09(1656.63,2284.46) | 1169292(948240,1423983) | 1577.90(1281.79,1915.68) | -0.70(-0.73,-0.68) | 60864(46979,77128) | 220.91(171.71,278.55) | 130262(98096,168253) | 175.94(132.04,227.57) | -0.72(-0.75,-0.69) |
| Timor-Leste | 4959(4081,5853) | 2148.28(1744.06,2568.74) | 12991(10649,15601) | 2108.02(1724.75,2532.93) | -0.22(-0.27,-0.16) | 553(413,714) | 235.77(177.66,302.54) | 1434(1076,1850) | 230.69(174.77,295.04) | -0.21(-0.26,-0.16) |
| Togo | 15561(12843,18613) | 1417.13(1156.35,1710.30) | 48971(40198,58563) | 1529.99(1244.56,1844.14) | 0.25(0.19,0.30) | 2347(1715,3054) | 210.57(156.11,272.08) | 7408(5385,9636) | 229.03(167.48,295.68) | 0.28(0.23,0.34) |
| Tonga | 798(662,946) | 2006.06(1651.89,2387.85) | 973(798,1179) | 1684.40(1381.03,2044.17) | -0.63(-0.66,-0.61) | 95(72,121) | 236.60(180.63,300.98) | 114(86,148) | 196.19(148.52,254.68) | -0.67(-0.70,-0.65) |
| Trinidad and Tobago | 4798(3734,6054) | 778.21(607.05,980.39) | 11528(9079,14452) | 867.90(682.36,1088.08) | 0.32(0.31,0.34) | 420(286,582) | 67.46(46.70,92.17) | 986(695,1333) | 74.44(52.16,101.03) | 0.29(0.27,0.31) |
| Tunisia | 73699(60622,87702) | 2009.66(1643.77,2404.31) | 224342(187541,264396) | 2432.12(2032.99,2864.02) | 0.68(0.65,0.72) | 7256(5431,9420) | 196.07(147.93,253.15) | 21756(16726,27602) | 235.37(181.05,298.48) | 0.67(0.63,0.71) |
| Turkmenistan | 20055(16202,24251) | 1399.99(1128.08,1703.43) | 31524(25375,38459) | 1111.29(894.55,1355.89) | -0.94(-1.05,-0.84) | 1830(1332,2422) | 125.90(93.19,164.78) | 2838(2044,3768) | 98.92(72.46,129.97) | -0.96(-1.06,-0.86) |
| Tuvalu | 96(79,113) | 2058.02(1676.60,2454.17) | 132(110,157) | 1808.17(1490.78,2168.00) | -0.51(-0.53,-0.48) | 11(9,15) | 244.95(186.31,315.14) | 15(12,20) | 211.42(159.99,271.34) | -0.54(-0.57,-0.52) |
| Türkiye | 604432(506812,709626) | 2359.09(1961.95,2796.45) | 1709343(1439819,1978102) | 2610.03(2200.99,3014.72) | 0.37(0.27,0.47) | 58254(44354,74914) | 226.16(173.36,290.51) | 163386(126016,205726) | 249.08(192.72,312.91) | 0.18(0.04,0.33) |
| Uganda | 82114(66640,99046) | 1538.01(1241.91,1866.76) | 198414(160968,238939) | 1548.66(1254.55,1874.57) | -0.10(-0.16,-0.04) | 11536(8429,15001) | 213.05(157.65,274.70) | 28173(20221,36954) | 216.15(158.54,281.46) | -0.07(-0.12,-0.01) |
| Ukraine | 849578(678091,1036218) | 1748.36(1400.11,2127.95) | 686810(547019,843316) | 1307.68(1044.24,1602.82) | -1.07(-1.11,-1.03) | 77723(57828,101298) | 160.04(118.77,208.74) | 61158(45070,79857) | 116.88(85.48,153.33) | -1.13(-1.17,-1.09) |
| United Arab Emirates | 11490(9847,13207) | 2036.42(1682.97,2417.47) | 113621(96564,133095) | 2336.61(1940.45,2781.67) | 0.36(0.32,0.40) | 1103(815,1447) | 194.09(146.89,250.52) | 10811(8015,14143) | 219.83(164.72,283.76) | 0.32(0.27,0.36) |
| United Kingdom | 1407257(1179120,1647832) | 2247.45(1882.17,2628.31) | 2101363(1776210,2421169) | 2318.45(1955.64,2678.25) | 0.17(0.10,0.24) | 114969(90701,141649) | 184.93(145.91,227.64) | 171826(136509,208849) | 191.62(152.08,233.16) | 0.18(0.09,0.27) |
| United Republic of Tanzania | 114602(93138,138370) | 1289.89(1046.77,1561.07) | 304058(247166,367331) | 1434.16(1166.52,1734.49) | 0.24(0.17,0.32) | 16209(11778,21275) | 179.94(132.96,233.06) | 43193(31579,56522) | 200.44(149.10,259.92) | 0.26(0.19,0.33) |
| United States of America | 6294685(5365449,7166913) | 2783.56(2371.74,3170.72) | 12171110(10998041,13239815) | 2955.77(2670.42,3219.62) | 0.29(0.14,0.44) | 684908(548632,829255) | 303.61(242.89,367.20) | 1302958(1088162,1529295) | 317.61(265.19,372.70) | 0.24(0.08,0.41) |
| Uruguay | 25855(20212,32165) | 948.72(741.94,1180.48) | 37163(29089,45948) | 958.90(752.83,1181.19) | 0.10(0.02,0.18) | 2133(1529,2854) | 78.46(55.89,105.22) | 3051(2227,4060) | 79.54(57.68,106.16) | 0.11(0.03,0.19) |
| Uzbekistan | 127471(103741,153469) | 1461.25(1186.05,1767.59) | 225558(180319,274894) | 1244.78(996.18,1520.39) | -0.54(-0.57,-0.51) | 11570(8486,15269) | 131.42(97.38,171.67) | 20284(14568,26776) | 110.35(80.67,144.05) | -0.58(-0.61,-0.55) |
| Vanuatu | 1056(888,1226) | 2221.69(1827.92,2623.47) | 2589(2178,3036) | 2002.78(1648.85,2385.15) | -0.36(-0.37,-0.35) | 124(95,159) | 259.03(198.59,328.45) | 305(232,391) | 234.01(177.97,299.81) | -0.35(-0.36,-0.34) |
| Venezuela (Bolivarian Republic of) | 71888(56126,90572) | 1001.33(785.65,1259.67) | 261294(207677,326468) | 1259.30(1002.07,1573.20) | 0.71(0.65,0.78) | 7125(4962,9702) | 97.37(69.79,129.91) | 25382(18591,33416) | 122.03(89.41,160.33) | 0.70(0.64,0.77) |
| Viet Nam | 493837(398085,602358) | 1668.05(1342.81,2036.94) | 1305763(1069007,1576819) | 1902.16(1554.60,2296.02) | 0.27(0.20,0.35) | 49467(36125,65264) | 166.24(122.81,217.84) | 131234(97546,169706) | 189.87(141.90,244.87) | 0.34(0.29,0.39) |
| Yemen | 81877(68076,95767) | 2157.49(1771.19,2560.15) | 237014(196132,281504) | 2153.53(1761.84,2581.38) | -0.03(-0.10,0.03) | 8040(6026,10348) | 209.86(159.14,268.11) | 23288(17249,30055) | 209.20(156.42,268.34) | -0.04(-0.11,0.03) |
| Zambia | 32213(25856,39020) | 1319.39(1056.73,1603.29) | 88377(71995,106384) | 1442.42(1171.88,1740.92) | 0.17(0.10,0.24) | 4553(3282,5995) | 182.73(134.36,238.86) | 12432(9098,16321) | 199.05(148.55,258.20) | 0.18(0.11,0.25) |
| Zimbabwe | 53321(42633,64906) | 1651.36(1323.27,2009.43) | 95467(76916,116245) | 1770.37(1426.87,2165.75) | 0.21(0.11,0.31) | 7743(5636,10211) | 237.04(175.49,308.40) | 13760(10087,18123) | 251.80(187.47,328.74) | 0.20(0.09,0.30) |

YLDs, years lived with disability; COPD, chronic obstructive pulmonary disease; ASR, age-standardised rate; EAPC, estimated annual percentage change; UI, uncertainty interval; CI, confidence interval

**Table S2.** Burden of prevalence and YLDs for COPD by sex at the global level in 2021.

|  | **Prevalence** | | | | **YLDs** | | | |
| --- | --- | --- | --- | --- | --- | --- | --- | --- |
|  | **Male** | | **Female** | | **Male** | | **Female** | |
| **Age group**  **(years)** | **Number**  **(95% UI)** | **ASR**  **per 100 000**  **(95% UI)** | **Number**  **(95% UI)** | **ASR per 100 000**  **(95% UI)** | **Number**  **(95% UI)** | **ASR**  **per 100 000**  **(95% UI)** | **Number**  **(95% UI)** | **ASR per 100 000**  **(95% UI)** |
| 15-19 | 548082(446827,638682) | 171.10(139.49,199.38) | 548880(465778,620255) | 180.76(153.39,204.27) | 74364(56982,91288) | 23.21(17.79,28.50) | 78612(61770,95376) | 25.89(20.34,31.41) |
| 20-24 | 751178(621190,880728) | 247.58(204.74,290.28) | 772361(654194,891789) | 262.93(222.70,303.59) | 101014(78392,123495) | 33.29(25.84,40.70) | 108922(85106,133508) | 37.08(28.97,45.45) |
| 25-29 | 995184(831733,1182021) | 334.68(279.71,397.51) | 1035923(860916,1202845) | 356.00(295.86,413.37) | 130867(102668,160804) | 44.01(34.53,54.08) | 143836(112948,175848) | 49.43(38.82,60.43) |
| 30-34 | 1302500(1093518,1527011) | 426.28(357.89,499.76) | 1355564(1144415,1582064) | 453.47(382.84,529.24) | 166320(132293,201002) | 54.43(43.30,65.78) | 186786(148047,228949) | 62.48(49.53,76.59) |
| 35-39 | 1503354(1282188,1756254) | 531.10(452.97,620.45) | 1549941(1319398,1791970) | 557.93(474.94,645.05) | 188797(150993,227245) | 66.70(53.34,80.28) | 209468(164751,256740) | 75.40(59.30,92.42) |
| 40-44 | 1808276(1586036,2062164) | 717.11(628.98,817.80) | 1816963(1579650,2054787) | 732.38(636.72,828.24) | 223793(182146,265082) | 88.75(72.23,105.12) | 241738(195520,290026) | 97.44(78.81,116.90) |
| 45-49 | 2897174(2418956,3373846) | 1218.01(1016.96,1418.40) | 2771098(2324502,3225984) | 1175.97(986.45,1369.02) | 353623(278129,434581) | 148.67(116.93,182.70) | 370588(289803,460492) | 157.27(122.98,195.42) |
| 50-54 | 4429811(3673007,5235690) | 1995.59(1654.65,2358.63) | 4143103(3447034,4888096) | 1858.37(1546.15,2192.54) | 528953(412810,652257) | 238.29(185.97,293.84) | 554067(427280,694800) | 248.52(191.65,311.65) |
| 55-59 | 5643726(4763293,6575129) | 2898.31(2446.17,3376.63) | 5402992(4526082,6334883) | 2688.01(2251.74,3151.63) | 656299(521524,798514) | 337.04(267.83,410.07) | 713195(545133,878570) | 354.82(271.21,437.09) |
| 60-64 | 6325157(5518532,7228206) | 4066.66(3548.05,4647.26) | 6206803(5388797,7028397) | 3772.88(3275.65,4272.30) | 723895(586691,867916) | 465.42(377.20,558.01) | 795194(632467,947148) | 483.37(384.45,575.74) |
| 65-69 | 7954367(6818283,9238425) | 6033.66(5171.90,7007.66) | 7956603(6842156,9112424) | 5525.08(4751.20,6327.68) | 897670(715543,1080785) | 680.91(542.76,819.81) | 1028509(816014,1264129) | 714.20(566.64,877.81) |
| 70-74 | 8287808(7026546,9621746) | 8598.08(7289.60,9981.95) | 8525741(7320100,9829551) | 7789.78(6688.21,8981.04) | 916764(721948,1101853) | 951.08(748.98,1143.10) | 1081289(843333,1320411) | 987.95(770.53,1206.43) |
| 75-79 | 6950130(5902355,7985652) | 11624.87(9872.35,13356.90) | 7632073(6523545,8791779) | 10585.74(9048.20,12194.26) | 753223(590779,915950) | 1259.85(988.14,1532.03) | 952337(746877,1164154) | 1320.90(1035.92,1614.69) |
| 80-84 | 5329337(4533182,6109674) | 14540.43(12368.22,16669.48) | 6732094(5759922,7673950) | 13218.02(11309.22,15067.29) | 554681(446307,682973) | 1513.38(1217.69,1863.41) | 805636(651641,977738) | 1581.81(1279.45,1919.72) |
| 85-89 | 3055683(2626966,3496282) | 17711.37(15226.44,20265.18) | 4629878(3942859,5320119) | 16262.80(13849.59,18687.32) | 308099(248381,370130) | 1785.80(1439.67,2145.35) | 531505(424965,642031) | 1866.95(1492.72,2255.18) |
| 90-94 | 1190777(1014928,1370072) | 20430.25(17413.20,23506.43) | 2277302(1964413,2645695) | 18881.73(16287.48,21936.18) | 115857(91634,140915) | 1987.77(1572.17,2417.68) | 244880(195744,296638) | 2030.36(1622.97,2459.51) |
| ≥95 | 353467(297107,416499) | 23376.82(19649.38,27545.46) | 836157(711219,977601) | 21231.60(18059.18,24823.12) | 33223(25826,40779) | 2197.25(1708.03,2696.97) | 83503(65631,103212) | 2120.31(1666.51,2620.76) |

YLDs, years lived with disability; COPD, chronic obstructive pulmonary disease; ASR, age-standardised rate; UI, uncertainty interval

**Table S3.** Burden of prevalence and YLDs for COPD by age at the global level in 1990 and 2021.

|  | **Prevalence** | | | | **YLDs** | | | |
| --- | --- | --- | --- | --- | --- | --- | --- | --- |
|  | **1990** | | **2021** | | **1990** | | **2021** | |
| **Age**  **group**  **(years)** | **Number**  **(95% UI)** | **ASR**  **per 100 000**  **(95% UI)** | **Number**  **(95% UI)** | **ASR per 100 000**  **(95% UI)** | **Number**  **(95% UI)** | **ASR**  **per 100 000**  **(95% UI)** | **Number**  **(95% UI)** | **ASR per 100 000**  **(95% UI)** |
| 15-19 | 923322(779685,1039855) | 177.76(150.24,200.12) | 1096962(912972,1255511) | 175.80(146.45,200.90) | 124263(98287,147438) | 23.92(18.95,28.35) | 152976(120674,183179) | 24.52(19.35,29.35) |
| 20-24 | 1300219(1097970,1480746) | 264.23(223.40,300.85) | 1523539(1286096,1768637) | 255.13(215.62,296.17) | 173130(137780,209020) | 35.18(28.02,42.43) | 209937(164314,254050) | 35.16(27.62,42.54) |
| 25-29 | 1599222(1345734,1849564) | 361.31(304.19,415.63) | 2031107(1701864,2378802) | 345.22(289.47,403.71) | 208823(166260,248767) | 47.18(37.73,56.19) | 274703(218295,331616) | 46.69(37.12,56.36) |
| 30-34 | 1784885(1541165,2034222) | 463.10(399.97,527.12) | 2658065(2253390,3091579) | 439.73(372.99,510.74) | 228705(184738,270023) | 59.34(47.93,69.95) | 353105(281798,422248) | 58.41(46.88,69.85) |
| 35-39 | 2006027(1735785,2276513) | 569.50(492.87,646.07) | 3053294(2628872,3521875) | 544.39(468.83,627.40) | 255477(206751,302492) | 72.53(58.80,85.60) | 398265(317561,477118) | 71.01(56.80,85.00) |
| 40-44 | 2165927(1918630,2419938) | 756.04(669.76,844.43) | 3625239(3198797,4080432) | 724.68(640.17,814.63) | 272213(226317,320131) | 95.02(79.07,111.74) | 465531(380734,546950) | 93.06(76.20,109.30) |
| 45-49 | 3010558(2483602,3538523) | 1296.56(1070.02,1522.36) | 5668271(4738077,6613069) | 1197.09(1001.22,1395.46) | 377884(296798,472980) | 162.74(128.22,203.43) | 724211(577427,897010) | 152.95(122.03,189.18) |
| 50-54 | 4422469(3666477,5199572) | 2080.46(1727.91,2443.34) | 8572914(7134366,10083735) | 1926.83(1605.69,2265.12) | 543875(423257,667529) | 255.86(199.79,313.05) | 1083020(843182,1336598) | 243.42(190.00,298.20) |
| 55-59 | 5474428(4633321,6341136) | 2955.96(2502.32,3423.02) | 11046718(9313929,12903118) | 2791.49(2357.26,3251.93) | 664811(529127,804127) | 358.97(286.54,433.27) | 1369494(1086989,1679194) | 346.07(275.79,423.76) |
| 60-64 | 6434864(5632301,7269949) | 4006.53(3507.39,4525.45) | 12531961(10925343,14197269) | 3915.65(3414.39,4435.51) | 763926(619339,905111) | 475.64(385.85,563.36) | 1519089(1231019,1797978) | 474.64(385.22,561.74) |
| 65-69 | 7208494(6175598,8382506) | 5831.67(5000.75,6776.45) | 15910970(13650291,18335833) | 5768.14(4959.64,6644.04) | 851117(679719,1021589) | 688.55(552.02,825.53) | 1926179(1543162,2289441) | 698.29(560.23,829.53) |
| 70-74 | 6902524(5868323,7913184) | 8153.12(6932.61,9342.63) | 16813550(14344051,19300269) | 8168.29(7004.99,9362.68) | 810190(648118,967287) | 956.98(766.00,1141.77) | 1998053(1588822,2394496) | 970.69(772.52,1162.79) |
| 75-79 | 6385702(5419313,7377027) | 10373.89(8808.33,11984.08) | 14582203(12506146,16709398) | 11056.81(9488.77,12666.67) | 714198(571018,858027) | 1160.25(928.90,1393.49) | 1705560(1357580,2043870) | 1293.22(1033.70,1546.94) |
| 80-84 | 4496882(3778908,5179744) | 12711.70(10690.69,14608.52) | 12061430(10284739,13827369) | 13771.42(11777.48,15735.88) | 483167(391083,575736) | 1365.81(1106.73,1626.60) | 1360316(1107600,1621453) | 1553.17(1265.15,1850.97) |
| 85-89 | 2275643(1923004,2640257) | 15059.44(12728.71,17440.45) | 7685561(6586064,8804588) | 16809.41(14429.55,19172.62) | 233917(185640,281182) | 1547.98(1229.65,1860.23) | 839603(679141,1012125) | 1836.33(1485.61,2213.24) |
| 90-94 | 738905(618511,868124) | 17243.23(14491.90,20192.28) | 3468079(2975176,3993270) | 19386.25(16668.16,22319.42) | 72400(57435,88192) | 1689.54(1341.48,2055.30) | 360737(291582,430668) | 2016.49(1631.00,2407.23) |
| ≥95 | 196340(165451,232252) | 19285.20(16254.09,22790.94) | 1189625(1013459,1383377) | 21826.74(18607.23,25366.19) | 18568(14636,22953) | 1823.78(1438.21,2251.64) | 116727(94448,141749) | 2141.65(1735.98,2599.97) |

YLDs, years lived with disability; COPD, chronic obstructive pulmonary disease; ASR, age-standardised rate; UI, uncertainty interval

**Table S4.** Projected trends of prevalence and YLDs for COPD from 2022 to 2050 at the global level.

|  | **Prevalence** | | **YLDs** | |
| --- | --- | --- | --- | --- |
| **Year** | **Number** | **ASR**  **per 100000** | **Number** | **ASR per 100000** |
| 2022 | 123099393 | 2038.04 | 14800412 | 245.04 |
| 2023 | 124461155 | 2035.12 | 14975684 | 244.87 |
| 2024 | 125810153 | 2031.99 | 15149765 | 244.69 |
| 2025 | 127140026 | 2028.66 | 15321842 | 244.48 |
| 2026 | 128460643 | 2025.37 | 15493945 | 244.28 |
| 2027 | 129755327 | 2021.98 | 15663359 | 244.08 |
| 2028 | 131024813 | 2018.42 | 15829507 | 243.85 |
| 2029 | 132270605 | 2014.67 | 15993065 | 243.60 |
| 2030 | 133485685 | 2010.78 | 16153268 | 243.33 |
| 2031 | 134680551 | 2007.02 | 16312615 | 243.09 |
| 2032 | 135850649 | 2003.23 | 16469905 | 242.86 |
| 2033 | 136936689 | 1999.33 | 16616906 | 242.61 |
| 2034 | 137985019 | 1995.33 | 16759462 | 242.35 |
| 2035 | 138997779 | 1991.29 | 16897956 | 242.08 |
| 2036 | 139988948 | 1987.45 | 17035389 | 241.85 |
| 2037 | 140946075 | 1983.66 | 17169818 | 241.65 |
| 2038 | 141863492 | 1979.84 | 17299404 | 241.43 |
| 2039 | 142740628 | 1976.01 | 17424183 | 241.21 |
| 2040 | 143578677 | 1972.24 | 17544351 | 240.99 |
| 2041 | 144389650 | 1968.69 | 17662575 | 240.82 |
| 2042 | 145163167 | 1965.22 | 17777183 | 240.67 |
| 2043 | 145896076 | 1961.76 | 17886822 | 240.51 |
| 2044 | 146592758 | 1958.35 | 17992112 | 240.36 |
| 2045 | 147257777 | 1955.07 | 18093725 | 240.22 |
| 2046 | 147900279 | 1952.02 | 18193778 | 240.12 |
| 2047 | 148511074 | 1949.06 | 18290733 | 240.05 |
| 2048 | 149088059 | 1946.15 | 18383522 | 239.97 |
| 2049 | 149636206 | 1943.36 | 18472824 | 239.91 |
| 2050 | 150159037 | 1940.70 | 18559113 | 239.86 |

YLDs, years lived with disability; COPD, chronic obstructive pulmonary disease; ASR,age-standardised rate

**Table S5.** Decomposition analysis of COPD prevalence from 1990 to 2021 at the global and regional levels.

| **Location** | **Overall**  **Difference** | **Aging** | **Population** | **Epidemiological Change** | **Aging**  **Percentage** | **Population Percentage** | **Epidemiological Change Percentage** |
| --- | --- | --- | --- | --- | --- | --- | --- |
| Global | 66193077.4 | 24061357.68 | 42172843.38 | -41123.67 | 36.35 | 63.71 | -0.06 |
| African Region | 466602.64 | -14342.56 | 461124.09 | 19821.11 | -3.07 | 98.83 | 4.25 |
| Eastern Mediterranean Region | 4497016.74 | 834056.76 | 3366885.71 | 296074.26 | 18.55 | 74.87 | 6.58 |
| European Region | 3649139.95 | 2350568.81 | 1366428.87 | -67857.73 | 64.41 | 37.45 | -1.86 |
| Region of the Americas | 424042.76 | 131404.72 | 253423.79 | 39214.25 | 30.99 | 59.76 | 9.25 |
| South-East Asia Region | 7179909.98 | 2615545.65 | 4530587.41 | 33776.92 | 36.43 | 63.1 | 0.47 |
| Western Pacific Region | 3312490.57 | 2804896.14 | 758145.65 | -250551.22 | 84.68 | 22.89 | -7.56 |
| World Bank High Income | 3949445.33 | 1787467.18 | 2135189.46 | 26788.69 | 45.26 | 54.06 | 0.68 |

COPD, chronic obstructive pulmonary disease

**Table S6.** Decomposition analysis of COPD YLDs from 1990 to 2021 at the global and regional levels.

| **Location** | **Overall**  **Difference** | **Aging** | **Population** | **Epidemiological**  **Change** | **Aging**  **Percentage** | **Population**  **Percentage** | **Epidemiological**  **Change Percentage** |
| --- | --- | --- | --- | --- | --- | --- | --- |
| Global | 8060840.57 | 2754049.18 | 5035338.08 | 271453.31 | 34.17 | 62.47 | 3.37 |
| African Region | 65870.73 | -1962.45 | 65336.36 | 2496.82 | -2.98 | 99.19 | 3.79 |
| Eastern Mediterranean Region | 531896.11 | 101633.01 | 418397.03 | 11866.07 | 19.11 | 78.66 | 2.23 |
| European Region | 321081.26 | 209338.92 | 123614.41 | -11872.07 | 65.2 | 38.5 | -3.7 |
| Region of the Americas | 36304.98 | 10907.55 | 21815.89 | 3581.54 | 30.04 | 60.09 | 9.87 |
| South-East Asia Region | 995978.78 | 356435.88 | 626789.22 | 12753.68 | 35.79 | 62.93 | 1.28 |
| Western Pacific Region | 438168.42 | 380501.89 | 102718.72 | -45052.19 | 86.84 | 23.44 | -10.28 |
| World Bank High Income | 383869.35 | 169156.69 | 204026.64 | 10686.02 | 44.07 | 53.15 | 2.78 |

COPD, chronic obstructive pulmonary disease; YLDs, years lived with disability

**3. Supplementary Figures**

**Fig. S1** Trends in the number of prevalent cases (A) and age-standardised prevalence rate (B) for chronic obstructive pulmonary disease (COPD) at the global and regional levels from 1990 to 2021. Prevalence estimates represent population-level indicators of potential rehabilitation needs.

**Fig. S2** Trends in the number of years lived with disability (YLDs) (A) and age-standardised years lived with disability rate (B) for chronic obstructive pulmonary disease (COPD) at the global and regional levels from 1990 to 2021.

**Fig. S3** Decomposition analysis of prevalent cases (A) and years lived with disability (YLDs) (B) for chronic obstructive pulmonary disease (COPD) at the global and regional levels from 1990 to 2021. Prevalence estimates represent population-level indicators of potential rehabilitation needs.
